# Nanoparticle-Mediated Photothermal Therapy Limitation in Clinical Applications Regarding Pain Management

**DOI:** 10.3390/nano12060922

**Published:** 2022-03-10

**Authors:** Marzieh Salimi, Sara Mosca, Benjamin Gardner, Francesca Palombo, Pavel Matousek, Nicholas Stone

**Affiliations:** 1 School of Physics and Astronomy, University of Exeter, Exeter EX4 4QL, UK; m.a.salimi@exeter.ac.uk (M.S.); b.gardner@exeter.ac.uk (B.G.); f.palombo@exeter.ac.uk (F.P.); 2 Central Laser Facility, Research Complex at Harwell, The Science and Technology Facilities Council Rutherford Appleton Laboratory, UK Research and Innovation, Didcot OX11 0QX, UK; sara.mosca@stfc.ac.uk

**Keywords:** nanoparticles, photothermal therapy, cancer, pain management, Raman spectroscopy

## Abstract

The development of new effective cancer treatment methods has attracted much attention, mainly due to the limited efficacy and considerable side effects of currently used cancer treatment methods such as radiation therapy and chemotherapy. Photothermal therapy based on the use of plasmonically resonant metallic nanoparticles has emerged as a promising technique to eradicate cancer cells selectively. In this method, plasmonic nanoparticles are first preferentially uptaken by a tumor and then selectively heated by exposure to laser radiation with a specific plasmonic resonant wavelength, to destroy the tumor whilst minimizing damage to adjacent normal tissue. However, several parameters can limit the effectiveness of photothermal therapy, resulting in insufficient heating and potentially leading to cancer recurrence. One of these parameters is the patient’s pain sensation during the treatment, if this is performed without use of anesthetic. Pain can restrict the level of applicable laser radiation, cause an interruption to the treatment course and, as such, affect its efficacy, as well as leading to a negative patient experience and consequential general population hesitancy to this type of therapy. Since having a comfortable and painless procedure is one of the important treatment goals in the clinic, along with its high effectiveness, and due to the relatively low number of studies devoted to this specific topic, we have compiled this review. Moreover, non-invasive and painless methods for temperature measurement during photothermal therapy (PTT), such as Raman spectroscopy and nanothermometry, will be discussed in the following. Here, we firstly outline the physical phenomena underlying the photothermal therapy, and then discuss studies devoted to photothermal cancer treatment concerning pain management and pathways for improved efficiency of photothermal therapy whilst minimizing pain experienced by the patient.

## 1. Introduction

Cancer is one of the biggest health problems around the world. In their lifetime, one in two people will develop the disease and one in three will die from it [1]. In 2020, 2.7 million people in the European Union were diagnosed with the disease, and another 1.3 million people lost their lives to it [2]. Furthermore, incidence and deaths are predicted to increase by more than 24% by 2035, making it the leading cause of death in the EU [3]. Due to the increasing cancer mortality, associated also with increasing population age, developing an effective therapeutic method has become a critical clinical priority. Surgical resection, radiation therapy, chemotherapy, and their combination are traditional cancer treatment approaches used in the clinic [4,5,6,7,8]. Radiation therapy and chemotherapy have been used to eradicate the cancer cells and inhibit them from spreading in the body (metastasis) after the surgical procedure [9,10]. However, these approaches have also significant drawbacks, such as irreversible damage to healthy tissues, limited therapeutic efficacy, and reduced patient’s quality of life. Finding an efficient and safe cancer treatment method with reduced side effects has therefore attracted considerable attention among researchers [5,6,11,12,13].

In recent years, increasing the temperature of tumor cells using external heat sources, e.g., ultrasound, microwaves, and lasers, is emerging as a promising technique for the treatment of different cancers [14,15,16]. In this area, in particular, photothermal therapy (PTT) has attracted major attention as a promising method due to its ability to eradicate cancer cells by delivering a specific amount of energy to the tumor [5,17,18,19]. In this approach, the temperature of tumor tissue is increased to 41–50 °C using a high-power near-infrared (NIR) laser [20,21]. Cancer cells are more sensitive to heat than normal cells due to the differential expression of heat shock proteins (HSPs) involved in the cellular defense system against different stressors such as heat. Therefore, cancer cells will irretrievably be damaged by these elevated temperatures due to protein denaturation [22,23,24,25,26,27,28]. These damages can then lead the cancer cells to apoptosis (≤45 °C) or necrosis (>50 °C). In other words, moderate (40–45 °C for 15–60 min) and high-temperature hyperthermia (>50 °C for 4–6 min) are the main thermal therapy programs in the clinic [29]. Furthermore, the temperature increase at the tumor site can sensitize the cells to other treatments, such as chemotherapy [30,31].

Since human tissues have a relatively high absorption coefficient in the visible range of the electromagnetic spectrum, the excitation wavelength used in PTT is typically chosen from within the “biological window”, a combination of the NIR-I (650–950 nm) and NIR-II spectral ranges (1000–1400 nm) [32,33,34], to increase the NIR laser penetration depth [21,31,35,36].

With insufficient heating, self-repairing mechanisms such as heat shock proteins (HSPs) can be activated in cancer cells during hyperthermia, and consequently PTT treatment efficacy can decline [37]. To inhibit these mechanisms, plasmonic nanoparticles (NPs) are often used in PTT to induce a sufficient and selective local temperature rise to the neighboring cancer cells [38]. Metallic plasmonic NPs used in PTT are the photothermal agents that can absorb laser energy and convert it into heat (photothermal conversion). These NPs should possess strong absorption and heat conversion efficiency in the NIR region [21,39,40]. Gold NPs (AuNPs) are the optimum candidates for PTT due to their high NIR absorption, tunable resonance, and high conversion efficiency. The absorption properties of AuNPs are determined by several parameters, such as size, shape, and surface coating [21,23].

Besides AuNPs, carbon-based nanomaterials (CBNs) such as carbon nanotubes (single and multi-walled), graphene-based NPs such as graphene oxide (GO), and graphene quantum dots (GQDs) have shown promising outcomes in PTT studies [41,42,43,44,45]. Carbon nanotubes’ electronic and optical properties depend on the diameter and the relative orientation of the graphene elemental hexagons with respect to the axis tube [46,47]. Graphene-based NPs also present a honeycomb lattice formed by a single-atom-thick layer of sp2 hybridized carbon atoms and are classified according to the oxygen content, number of layers in the sheet, or their chemical composition [48]. The GQDs are zero-dimensional carbon nanomaterials holding significant molar extinction coefficients and broad absorption in the NIR region [45].

It is worth mentioning that the structure of carbon-based nanomaterials affects their in vivo behavior and anticancer photothermal effect. It is shown that graphene nanosheets circulate 2.2 times longer in the blood and accumulate more in the tumor compared to the single-walled carbon nanotubes (SWCNT). The photothermal conversion effect of GO depends on their concentration and light dose [49]. In addition, the cytotoxicity of GO is extremely low and can also decrease the toxicity of gold nanorods [50]. 

PTT using plasmonic NPs possesses high selectivity towards cancer over normal cells [17,35,51]. Additionally, PTT provides reduced invasiveness and systemic toxicity, shorter hospitalization, negligible side effects, and lower financial costs when compared to chemotherapy and radiation therapy [40,52,53,54,55,56]. 

Despite these benefits, a patient’s pain can still be a critical factor limiting the PTT applicability in clinical settings. The continuous conversion of NIR energy can induce overheating and damage to the normal tissues close to the tumor, causing inflammation and pain perceived by the patient [57]. Moreover, to achieve complete ablation of tumor cells, a high-power laser may be needed to increase the tumor temperature over 50 °C, which high temperature can be painful for the patient [58,59].

Other parameters that can indirectly cause pain in patients during PTT procedures include insufficient intracellular AuNP delivery, the random intracellular distribution of AuNPs, and their low laser−heat conversion efficacy [60].

The International Association for the Study of Pain (IASP) defines pain (accepted also by the World Health Organization (WHO)) as a subjective unpleasant sensory and emotional experience associated with actual or potential tissue damage [61,62]. Although future updates have been made to the list of associated pain terms, the IASP definition of pain itself has remained unchanged [63,64,65,66,67]. Based on established pain terminology, experiences that appear similar to pain but are not unpleasant, such as pricking, should not be classified as pain. On the other hand, unpleasant abnormal experiences (dysesthesia) are not necessarily pain since they might not have the typical sensory qualities of pain. 

Many people report pain in the absence of tissue damage or pathophysiological causes due to psychological reasons; for instance, pulsed infrared laser exposure can elicit compound nerve and muscle action potentials with resultant muscle contraction (optical nerve stimulation) [68,69]. It is worth mentioning that there is no method to distinguish patients’ experience from that due to tissue damage in the case of subjective reports. If patients consider their experience as pain and report it in the same way as pain caused by tissue damage, it should be accepted as pain since the definition has not tied pain to the specific stimulus [63].

To clarify pain terminologies and the underlying physiological phenomena, in Table 1, we list the most commonly used terminologies, including noxious stimulus, nociceptor, and nociceptive stimulus [70]. Briefly, a noxious stimulus is a stimulus that damages normal tissues and causes pain. Some types of tissue damage are not detected by any sensory receptors and hence do not cause pain. Therefore, not all noxious stimuli are adequate stimuli of nociceptors that are sensitive receptors to the noxious stimulus. The adequate stimuli of nociceptors are termed ‘‘nociceptive stimuli”, a subset of noxious stimuli that are tissue-damaging events transduced and encoded by nociceptors. Non-nociceptive receptors (e.g., tactile receptors, hot/cold receptors) may respond to noxious stimuli (e.g., mechanical or thermal, respectively) when these stimuli are above their particular thresholds. However, only nociceptors can encode the relevant properties of those stimuli (e.g., sharpness, heat intensity in the painful range) [70,71].

Since pain is a crucial parameter restricting the PTT clinical applications and, to the best of our knowledge, there is no comprehensive review study on pain management during PTT, this literature evaluation was carried out to overview different pain management strategies in PTT. To this end, we reviewed all clinical and animal PTT studies in which authors managed pain during the treatment application. The main keywords for the literature review were *photothermal therapy*, *laser thermotherapy*, *complications*, and *pain*. Most studies reporting pain management in patients during laser interstitial hyperthermia (without plasmonic nanoparticles) were performed in the 1990s. Regarding the similarities between laser interstitial hyperthermia and PTT (nanoparticles involved), the strategies of pain management applied in these studies were considered potential pain management methods in the future human PTT.

It should be noted that the principal pain management strategy during the PTT procedure remains (local) anesthesia, as with other clinical procedures. However, it is not always possible or desirable to employ this approach for clinical or other practical reasons (cost, availability). Different side effects and risks have been reported during pediatric anesthesia, such as asthma, upper respiratory tract infection, and cardiac arrest [72]. Moreover, anesthesia risks might be higher in patients with diabetes, high blood pressure, kidney problems, obstructive sleep apnea, and heart disease [73]. Wernli et al. showed that the overall risks of adverse outcomes within 30 days following coloscopy were higher among patients who received anesthesia than those who did not indicate the use of anesthesia services [74]. Regarding anesthesia drawbacks, other pain management strategies become critically important in a PTT procedure. On the other hand, traditional pain relief medication can also be applied for post-treatment pain relief. The pain management strategies mentioned in this review are the methods that have been used in studies as well as traditional pain relief medication.

### Principle of Photothermal Therapy Using AuNPs

To mitigate and manage the pain during PTT, it is required to increase the treatment efficiency by optimizing the parameters involved in the nanoparticles’ laser–heat conversion efficiency during laser exposure. To this end, the physical phenomena underlying the laser–heat conversion efficiency of plasmonic NPs will be discussed in this section. In the following part, effective parameters that can modify the conversion efficiency are discussed.

The lack of selectivity and the requirement of a high-power laser can be mitigated by using plasmonic NPs such as AuNPs in mediating PPT [75,76]. In other words, plasmonic NPs with high laser–heat conversion efficiency can selectively be uptaken by cancer tumors, most commonly due to the presence of a leaky vasculature, and under NIR laser irradiation (700–1000 nm), only cancerous tissue is heated above the damage threshold [77,78]. Consequently, PTT causes intracellular effects such as DNA damage and destruction of aberrant functional proteins in cancer cells [46,79]. Furthermore, rapid AuNP heating due to high-energy irradiation leads to cellular necrosis, which can trigger the release of cellular waste and damage-associated molecular patterns (DAMPs). On the other hand, low-power PTT can induce cellular apoptosis, triggering antitumor immunogenic responses [80,81].

**Figure 1:**
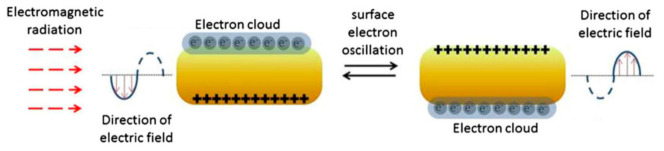
Schematic diagram of the localized surface plasmon resonance due to collective oscillations of conduction electrons at the surface of a gold nanorod in response to laser irradiation. Since the nanorod possesses two axes, a long and a short axis, the oscillations of the electron cloud can happen along both axes. Adapted with permission from [82].

AuNPs can convert NIR light into heat, on a picosecond timescale, due to their unique optical property called “localized surface plasmon resonance” (LSPR), which originates from the collective oscillations of conduction electrons at the surface (surface plasmon) in response to a specific laser wavelength, resulting in amplified laser absorption and scattering (Figure 1) [82]. The LSPR wavelength strongly depends on AuNPs’ properties, which can be optimized and tuned to ensure the highest laser–heat conversion required to reach the optimum temperature in the cancer tissues [83,84,85,86]. 

In detail, the amount of laser power that is absorbed by a single AuNP is dependent on its absorption efficiency, a_eff_, i.e., the ratio between absorption cross-section, σ_abs_, and extinction cross-section, σ_ext_:

σ_ext_ = σ_abs_ + σ_scat_, (1)

and

a_eff_ = σ_abs_/σ_ext_, (2)

where σ_scat_ is the scattering cross-section of the AuNP (m^2^) [35]. When an AuNP absorbs laser photon energy, a nonradiative process can take place, resulting in the dissipation of the absorbed energy into heat and inducing a temperature increase in the AuNP and its surroundings, called the photothermal effect [87]. During this process, the oscillating surface electrons transfer their kinetic energy into the gold nanoparticle lattice through electron−phonon interactions within a timescale of 2–5 ps, followed by phonon−phonon interactions with the surrounding medium within 100–380 ps [88,89]. 

Finally, heat dissipates through particle−medium interfaces at a rate that depends on the medium and particle size. The photothermal conversion efficiency of AuNPs strongly depends on several parameters, including plasmon resonance wavelength, incident radiation wavelength, AuNP size, shape and morphology, surface coating, and assembly state [90,91,92].

Photothermal conversion efficiency is defined as converting absorbed light energy into localized heat and usually mentioned as a percentage (Table 2) [93]. Plasmonic NPs possess a wide conversion efficiency range in the NIR-I and NIR-II regions [83,92,94,95,96,97,98,99,100,101,102,103,104,105,106,107,108,109]. Table 2 lists reported conversion efficiencies for several nanostructures regarding the most influential parameters, such as laser frequency, power, and NP size. The available PTT coupling agents mainly comprise metal nanoparticles (Au, Ag, Pd, and Ge), semiconductor nanoparticles, and carbon-based nanomaterials (carbon nanotubes and graphene). Gold-based nanoparticles display strong NIR absorption properties and consequently heat conversion efficiencies as high as 80% [110,111]; for instance, the conversion efficiency of Au nanorods at 809 nm ranges from 13% to 96% and decreases as the effective radius increases [92]. 

## 2. Pain Management in PTT and Laser Interstitial Hyperthermia Treatments 

### 2.1. Hepatic Cancer

Christian et al. in 1992 performed ultrasound (US)-guided interstitial hyperthermia using a neodymium-doped yttrium aluminum garnet (Nd:YAG) laser with a diffuser tip inserted in solid tissue followed by irradiation within the center of the tumor. Laser treatment was performed on liver metastases in eleven patients. A dedicated multi-puncture needle guide mountable on the transducer was designed for simultaneous laser coagulation and temperature registration. The needle guide possessed ten parallel canals, each separated by 5 mm. Thus, this steering device allowed accurate and simultaneous placement of both “laser fiber needles” and “temperature needles” within the same image plane. Laser coagulation was performed using a continuous-wave Nd:YAG laser with a 1064 nm wavelength [112]. Interstitial temperature monitoring using “temperature needles” via a dedicated multi-puncture needle guide was used during treatment. The laser treatment continued until the temperature display showed 60 °C or stayed constant at 45 °C for 15 min. The laser output was between 4 and 8 W; the exposure time varied between 5 and 45 min, depending on the size and vascularity of the metastases.

No severe complications were reported as a result of the treatment. Five patients had no complications at all; three patients experienced temporary minor pain, two patients had a slight increase in body temperature, and one patient developed a small pleural effusion and pneumoperitoneum, both resolving without any therapy.

Amin et al. in 1993 treated fifty five liver metastases in 21 patients using interstitial laser photocoagulation (ILP). Tumors were irradiated with an Nd:YAG laser via optical fibers passed through 19-gauge needles inserted under ultrasound (US) guidance. After ILP, dynamic computed tomography (CT) showed laser-induced necrosis as a new area of nonenhancement [113]. The maximum power was 2 W with an exposure time of 500 s. They did not apply any thermometry during the treatment. The heating of the tumor was evident in real-time US as an expanding and coalescing echogenic zone around the needle tips. 

Several complications were reported, including severe pain in four cases, and persistent pain for up to 10 days in 11 cases. Additional analgesia was often required for lesions just under the liver capsule, either for shoulder pain due to diaphragmatic irritation or abdominal and back pain thought to be due to heat conduction to nearby peritoneum or retroperitoneum. In four cases (two tumors adjacent to the diaphragm and two next to the peritoneum), the pain shortened the treatment time with intermittent bouts of 100–300 s rather than continuous 500 s.

In another study, Masters et al. in 1992 [114] treated ten patients with a total of 18 hepatic metastases with interstitial laser hyperthermia using an Nd:YAG laser to achieve an overall objective response rate of 44%. The procedure was performed under a combination of intravenous sedation and analgesia (Diazepam 5–10 mg and Pethidine 50–75 mg) together with a 24 h regimen of intravenous prophylactic antibiotics (Flucloxacillin and Gentamicin). Only one patient complained of shoulder tip pain, probably due to stimulation of the diaphragmatic peritoneum when treating a lesion high up under the dome of the diaphragm. Most patients described mild abdominal wall discomfort at the needle puncture sites; however, this resolved spontaneously within 48–72 h of treatment in most instances, and rarely required more than mild oral analgesia for relief. 

The evolving thermal changes occurring at the treatment site were monitored in real time using US (3.5 MHz). A laser power of 1.5 to 2 W per fiber was applied for at least 500 s for each metastasis.

Vogl et al. in 2002 reported a magnetic resonance imaging (MRI)-guided laser-induced thermotherapy (LITT) for 899 patients with malignant liver tumors [115]. A single dose of opioids (e.g., piritramide and pethidine) was administered intravenously for pain management during and after the treatment procedure. Furthermore, antiemetic medication such as metoclopramide and post-treatment oral pain medications such as metamizole and tramadol were prescribed eight hours after LITT as needed. Patients regularly reported having pain in the epigastric region during the following three weeks. The most frequent minor complication during or after LITT was pain at the local site of catheter insertion.

The safety parameters considered before, during, and after treatment are listed in Table 3. After treatment, all patients were instructed to take their body temperature twice daily for eight days following LITT. Patients with a body temperature higher than 38.4 °C for two days or longer took antibiotics orally for ten days, and then ultrasonography was performed 14 days after LITT. 

### 2.2. Breast Cancer

Harries et al. in 1994 used interstitial laser photocoagulation to treat forty-four patients with small breast cancers using a diode laser (805 nm) under local anesthesia. A needle was inserted aseptically into the tumor, and then the position of its tip in the center of the lesion was checked using ultrasonography. The needle was withdrawn by approximately 5 mm so that the tip of the fiber lay bare within the tumor. Subsequently, the lesion was treated with a relatively low-power laser at 2–3 W and exposure time of 500–750 s. Treatment was stopped prematurely in three patients at 200, 360, and 540 s because of pain; otherwise, all patients tolerated the treatment without serious complications [116]. They did not monitor the temperature during the treatment; only ultrasonography or CT monitored laser effects (necrosis) within the tumor in real time.

Robinson et al. in 1997 investigated the preclinical development of a laser fiberoptic interstitial delivery system for the thermal destruction of small breast cancers. They used a 60 W continuous-wave Nd:YAG laser (Fiber Tome, Dornier Medical Systems, Munich, Germany) with a fiberoptic delivery system that provided the power using Dornier’s light guide protection system mode. The fiber tip was optically prevented from overheating to avoid carbonization of the surrounding tissues. Laser irradiation started at 20 W for 30 s, decreased to 15 W for 30 s, then to 10 W for 30 s, and finally to 7 W for 90 s (3-min cycle) and 270 s (6-min cycle). The total calculated energy delivery was 1980 J in the 3-min cycle and 3240 J in the 6-min cycle [117]. Temperatures in the tissue were measured by eight separate needles, thermocouple probes (50-mm long, 23-gauge, type-T copper/constantan, Physitemp MT-23/5, Physitemp Instruments, Inc., Clifton, NJ, USA). Each thermocouple was calibrated against a precision thermometer. 

The temperature increased to 50–54 °C during the whole exposure period, and the highest registered temperature was 56 °C during laser exposure.

They tested their system on pigs, and postoperative pain control was initiated by an intramuscular injection of buprenorphine hydrochloride (0.01 mg/kg) and maintained with fentanyl transdermal patches (2.5 mg fentanyl transdermal system in 0.1 mL alcohol USP) at a rate of 25 mg/h for 72 h, placed on the lateral aspect of the animal’s ear.

**Figure 2:**
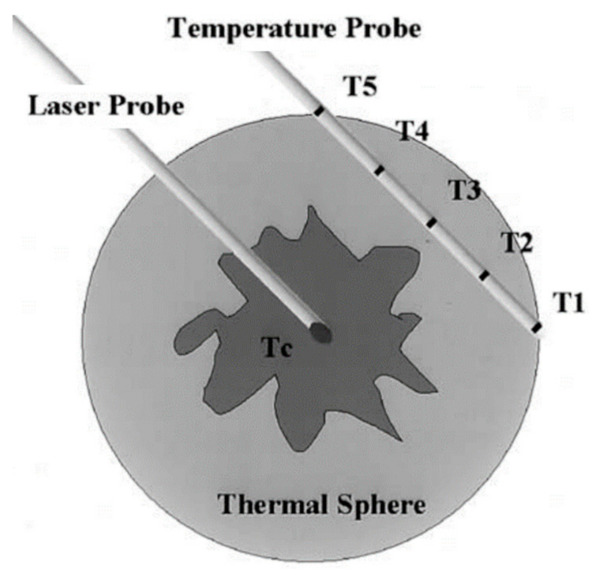
Schematic of laser and a temperature probes within and adjacent to the breast tumor. T1–T5 are peripheral thermal sensors. Adapted with permission from [118].

Dowlatshahi et al. in 2002 treated fifty-four patients with breast cancer using a stereotactically guided 805 nm laser beam via fiber in a 16G needle inserted into the tumor [118]. The average treatment time was 30 min. Five peripheral thermal sensors (T1-T5) on the needle adjacent to the tumor displayed real-time temperature, indicating treatment adequacy. The peripheral sensors showed the temperature increase while the central temperature (Tc) reached them from the laser probe at the center of the tumor (Figure 2). The treatment was terminated when all peripheral sensors recorded 60 °C.

Patients might experience pain during treatment if the initial field anesthesia was inadequate, mandating additional injection of bupivacaine (nerve block). The skin overlying the tumor was optionally cooled with a coolant spray in cases where the tumor was less than one cm from the skin. After the treatment, the needles were removed, the breast was decompressed, a light dressing was applied, and after one hour of observation, the patient was discharged home with oral analgesics and an ice pack on the breast [118]. 

### 2.3. Prostate Cancer

Rastinehad et al. in 2019 reported a clinical trial in which laser-excited gold–silica nanoshells (GSNs) were used in combination with magnetic resonance–ultrasound fusion imaging to ablate prostate tumors in 16 patients focally. They used AuroLase Therapy (Nanospectra Biosciences, Inc., Houston, TX, USA) (https://nanospectra.com/technology/ last accessed on 26 October 2021), which is a focal ablation modality that relies on laser excitation of GSNs to selectively target and treat focal lesions within the prostate [119]. 

**Figure 3:**
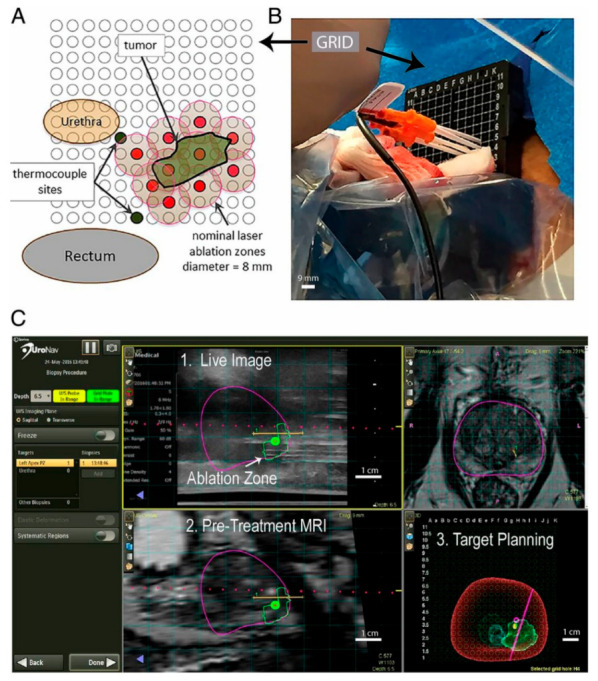
Photothermal treatment applied to a prostate tumor using the transperineal approach. (**A**) Axial view of the prostate ablation zone and the adjacent urethra and rectum overlaid with a rectangular transperineal grid (3-mm spacing). The introducer trocars (red) penetrated the ablation zone through the targeting grid, allowing for the 4 to 5 mm treatment radius (tan). (**B**) Laser introducers (orange hub) placed with the thermocouple (black) through the transperineal grid. (**C**) UroNav MR/US Fusion guidance for trocar placement with real-time ultrasound imaging (Scale bar: B, 9 mm; C, 1 cm). Adapted with permission from [119].

Near-infrared laser (810 ± 10 nm) light was delivered via a dual-lumen, water-cooled catheter, housing either a 10 mm optical fiber diffuser (OFD) and power up to 4.5 W or an 18 mm OFD with power up to 6.5 W. Needle thermocouples were placed near the urethra, urinary sphincter, and rectal wall to monitor and minimize the risk of tissue damage near critical structures. Patients subsequently underwent laser illumination under general anesthesia while positioned in the dorsal lithotomy position (Figure 3). 

The periprostatic nerve block was performed using 1% lidocaine without epinephrine under transrectal ultrasound guidance via the perineum. The procedure was performed with continuous cooling irrigation via a 16 or 18 French 3-way urinary catheter. Regarding the pain, one patient experienced transient substernal epigastric pain during the GSN infusion, which was attributed to the cold temperature of the GSN suspension taken directly from the storage refrigerator.

### 2.4. Head and Neck Cancer 

A clinical trial, which was last updated in February 2017, investigated PTT using AuroShell (TM) particles in patients with refractory and recurrent tumors for head and neck cancer [120]. Three treatment groups of five patients each were enrolled and observed for six months following photothermal treatment. Each group had received a single dose of AuroShell (TM) particles, which are silica–gold nanoshells coated with PEG (31), followed by one or more interstitial exposure using an 808 nm laser. Particle dose and laser power were increased in each dosing group; three different groups in this study included AuroShell-3.5, AuroShell-4.5, and AuroShell-5.0. The first group was treated with the lowest level of 4.5 mL/kg AuroShell particles concentrated to 100 optical density (OD) and 3.5 W laser power. The second and third groups were treated with up to 7.5 mL/kg AuroShell particles concentrated to 100 OD using 4.5 and 5 W laser power, respectively.

All adverse events were reported regardless of relation to photothermal treatment. The updated results showed that patients in the AuroShell-3.5 group had the experience of flushing, hypoxia, hypertension, gastroesophageal reflux, chills, and neoplasm-related pain. Patients in the AuroShell-4.5 group experienced hypertension, ptyalism, urinary tract infection, dehydration, muscle spasm, erythema, and generalized pain. In the AuroShell-5.0 group, only one patient did not experience any of these complications and pain.

## 3. Skin Pain following Laser Radiation 

Pain, usually represented as “stinging”, “burning”, or “prickling”, is possibly the most common side effect of photodynamic therapy (PDT) in the clinic, and it affects patient compliance and the acceptance of treatment modality [121,122,123]. Similarly, pain sensation due to different reasons, such as skin burning, can be one of the limiting challenges during PTT. For both superficial and deep tumors, the first barrier in front of the laser is the skin surface. In this regard, the protection of skin from burning during the photothermal treatment is important. 

Nociceptors located in the deeper skin layers are stimulated only via heat conduction. Thus, high stimulus intensities and skin surface temperatures above 60 °C are required in order to produce intense pain, which might already damage the stratum corneum (i.e., epidermis). The average depth of nociceptive terminals is 200 µm, with a range of 20 to 570 µm [124,125]. 

Moreover, it is essential to combine laser irradiation with water cooling of the irradiated body surface where sensitive pain receptors are very close to the body surface [126,127,128]. This surface cooling is expected to significantly decrease the pain (and associated tissue damage) accompanied by intense heating of a thin surface layer, especially in breast cancer treatment. Moreover, the pain associated with the photothermal treatment procedure might be diminished by the appropriate choice of irradiation parameters such as power, intensity, fluence, and irradiation area [129]. Other strategies for skin pain management in the clinic include topical pre-medication with lidocaine, prilocaine gel, injection of topical anesthetics, cooling with cold air, applying nerve blocks, and pulsed laser irradiation [130,131,132,133,134].

Regarding nerve blocking, A-delta (Aδ) nerve fibers would be suitable candidates as the first generators of the pain caused by laser irradiation. These fibers, found in the dermis but not in the epidermis, are involved in the pain induced by skin overheating (more than 42 °C at physiological conditions) [135,136].

Li et al. performed a preliminary clinical study on the treatment of (eleven patients) metastatic melanoma using photoimmunotherapy (ISPI), which combines selective photothermal therapy and immunological stimulation [137]. An 805 nm laser irradiation (1 W/cm^2^) was applied for 10 min on each tumor along with topical applications of imiquimod (immune response modifier). Local anesthetic using lidocaine 1% with adrenaline was administered for pain management; before laser irradiation, hypopigmented and non-pigmented melanoma nodules were injected with a 0.25% indocyanine green (ICG) dose of 0.5 mL/cm^3^ to increase the tumor absorption.

Furthermore, significant pain with the laser treatment was seen in around 20% of patients during the first treatment cycle, which was usually removed with oral premedication with narcotics, although one patient needed conscious sedation. The most frequently reported adverse events were rash (90.9%), pruritus (81.8%), and pain (54.5%).

**Figure 4:**
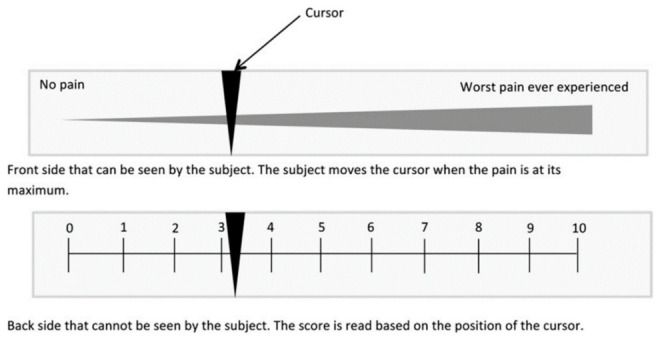
Visual analogical pain scale used for pain measurement during treatment. Reproduced with permission from [138].

On the other hand, measurement of the pain that patients experience during (or after) laser exposure and PTT is complex, and this can be due to the subjective characteristics of the pain. Patients feel pain based on their physiological and psychological aspects, and consequently their pain tolerance is entirely different [138]. The visual analogical pain scale is a method to score and evidence patients’ pain during treatment. In this method, the maximal pain experienced by a patient is quantified with the aid of a visual analog scale (VAS), as reported by Johnson and Langley and Sheppeard [139,140]. On this scale, the pain feeling is classified from 1 to 10 (Figure 4). For each site, the patients were asked to rank their most intense pain feeling during the illumination between 1 and 10.

Barge et al. applied the visual analogical pain scale to measure the pain in patients during laser irradiation in PDT [138]. Their results showed an increase in pain while increasing the light radiation power. The mean pain score was 0 in the absence of light. The mean pain score at the irradiance of 100 mW/cm^2^ was 3.35, and it reached a value of 6.63 at the irradiance of 180 mW/cm^2^ [138]. In this study, the light delivered to the skin had a uniform distribution, as measured using a light distributor. The authors did not mention the light dosimetry or the laser beam temporal properties (continuous wave or pulsed); since the used irradiance (180 mW/cm^2^) was even lower than MPE, feeling the pain with this intensity was unexpected [141]. The pain sensation in this study was due to the follicular fluorescence of the used PDT sensitizers. On the other hand, the pain induced by PDT mainly results from the hypersensitization of the nerve fibers involved in the pain response to overheating [122]. This PDT-induced hypersensitivity would then generate burning sensations even at physiological temperatures.

It is worth mentioning that the VAS method provides a measurement at discrete points during the treatment and not a record of the pain encountered along with the entire treatment course. 

## 4. Other Strategies for Pain Management in PTT

The very first strategy to mitigate pain in PTT would be to use the lowest temperatures able to provide the desired treatment effect (ideally ≤45 °C) [142]. However, under mild temperature increases, immunologic responses in the tumor decrease cancer cell death and elevate the risk of cancer recurrence, which is undesirable. There is no evidence that localized NP heating to a particular temperature is less painful than heating to the same temperature without NPs, but reaching an acceptable therapeutic temperature in the presence of NPs would need less exposure power and intensity than the situation without NP administration. Higher nanoparticle conversion efficiency means less laser irradiation and, consequently, less pain due to skin burning or healthy tissue damage during the photothermal treatment. 

Accordingly, to compensate for the attenuated therapeutic effects of using as low a laser irradiation energy as practical and to boost the PTT efficacy, the development of photothermal nanostructures for more efficient NIR laser energy-to-heat conversion, especially with enhanced cellular uptake in cancer cells and localized accumulation in heat-hypersensitive subcellular organelles, is of great importance [143]. Hence, developing novel nuclear targeting photothermal agents with efficient photothermal conversion properties, acceptable biocompatibility, simple fabrication, and particularly intranuclear accumulation is highly desired for subcellular targeted PTT with considerably lower NIR laser exposure [60].

For effective tumor treatment, increasing the local temperature above 50 °C is necessary to reach the total necrosis of cancer cells. The cell apoptosis induced by lower heating (i.e., mild PTT such as at 45 °C) might be repaired through the assistance of tumor HSPs [60,142,144]. An efficient tumor-killing approach has been reported using graphene oxide loaded with SNX2112 (HSP90 inhibitor) and folic acid for ultrafast low-temperature PTT (LTPTT, 38–43 °C). Overactivated autophagy induced by ultrafast LTPTT led to the direct apoptosis of tumors and enabled the functional recovery of T cells to promote natural immunity for actively participating in the attack against tumors [12]. In other words, LTPTT is a relatively rapid antitumor method closely related to autophagy. Moreover, PTT has been combined with autophagy-regulating (overactivation or inhibition) drugs to achieve efficient tumor elimination [145,146].

**Figure 5:**
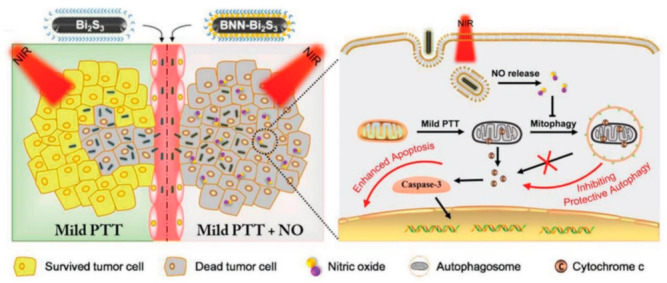
Autophagy is a prosurvival pathway in mild PTT, which can demolish the damaged mitochondria, reduce the release of cytochrome c, and inhibit the activity of caspase-3. NO downregulates the autophagy level to decrease its effects and triggers cellular apoptosis. Reproduced with permission from [151].

Another approach to improve the therapeutic efficacy of PTT is to combine mild PTT with other treatment modalities (synergistic effect) [147]. Co-administration of mild PTT and chemical drugs such as nitric oxide (NO) can significantly increase the antitumoral effect [148]. NO can inhibit the subcellular degraded processes, including autophagy, to accelerate cellular apoptosis [149]. Additionally, a relatively high NO concentration can directly cause cell death [150]. Zhang et al. reported nanocomposites based on bismuth sulfide (Bi_2_S_3_) nanoparticles and a hydrophobic NO donor (BNN), which was loaded on the surface of Bi_2_S_3_ nanoparticles to use the synergistic effect of both treatments (Figure 5) [151]. Consequently, the NIR laser triggered NO release from nanocomposites for sensitizing cancer cells towards photothermal therapy. Mild PTT enhances drug diffusion and accumulation in the tumor region, and the chemo-drugs compensate for inadequate heat damage [152].

**Figure 6:**
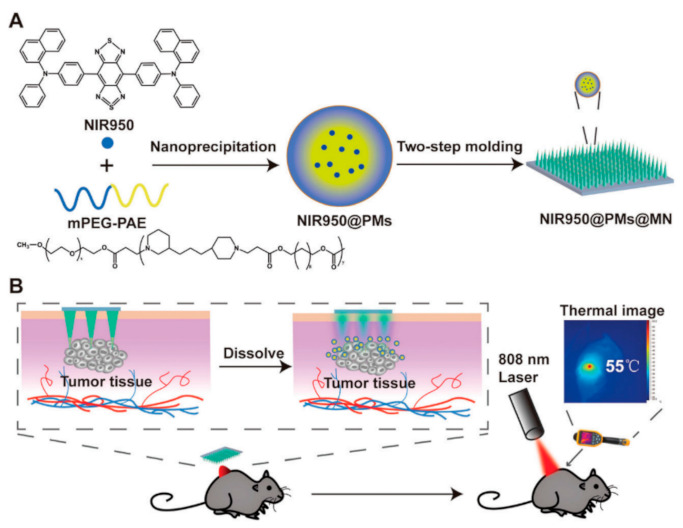
(**A**) Schematic illustration of the synthesis process of NIR950@PMs and NIR950@PMs@MN. (**B**) Schematic illustration of NIR950@PMs@MN application method in the photothermal treatment of tumor-bearing mice. Reproduced with permission from [153].

As mentioned before, the accumulation of photothermal NPs in the tumor cells is one of the most critical factors in increasing the PTT light-to-heat conversion efficiency. To this end, some novel approaches have been developed to increase NP delivery to the desired locations. Wei et al. introduced a dissolving microneedle (MN) system loaded with AIEgen (NIR950) for topical administration to treat malignant skin tumor melanoma in PTT [153]. In this study, NIR950-loaded polymeric micelles (NIR950@PMs) were prepared, and then micelles were concentrated on needle tips of MN (NIR950@PMs@MN). Their results showed that MN, NIR950@PMs could rapidly accumulate at the tumor site and reach an appropriate temperature for demolishing the cancer cells using only one-time laser irradiation (Figure 6).

The microneedle system has some advantages, such as improving the accumulation of photothermal NPs at the tumor site to reduce drug loss and systemic side effects, achieving the three-dimensional drug distribution due to the well-designed MN array structure, and increasing patients’ comfort. Microneedles can also relieve the pain and fear caused by traditional injection in the patient. Moreover, MNs can avoid unnecessary pain as their length does not reach the deep dermis layer in which sensory nerve endings are located, yielding less invasive and painless drug delivery [154,155]. It is also noteworthy that the best advantage of MN convenient application is encouraging as they might not need professional management training [156]. MNs are safer and more efficient than traditional intravenous or intratumoral injections as a new drug delivery platform.

As forementioned, heating of the tumor might cause an adverse protective reaction of the thermal regulation system with an increase in blood perfusion near the tumor and release of HSPs, which play an essential role in the cellular thermoresistance [157]. One solution would be using periodic heating with a single heating duration of around 10–15 s, which decreases the blood flow variation [158]. Another solution would be using indirect heating based on the laser irradiation of nearby tissues as an alternative to the direct irradiation of superficial tumors [159]. 

In this regard, two different strategies have been proposed for indirect tumor irradiation; first, the AuNPs are embedded in a spherical region around the tumor tissue and then exposed to the laser. Accordingly, a hot spherical region will appear in the healthy tissue, conducting heat from all sides into the tumor. This method is suitable in a tumor with a volume smaller than the volume of the hot ring; also, delivering the AuNPs in the spherical region and controlling their distribution might be complex. The second method is laser irradiation in a circular region around the tumor without NP administration. In this case, laser energy is scattered in all directions and absorbed mainly in the tumor because of the higher absorption coefficient of the tumor tissue. 

Consequently, the best approach for indirect tumor irradiation would be the combination of the above approaches and using a low volume fraction of AuNPs, mainly at the tumor periphery, to increase the local absorption of laser radiation scattered by the neighboring healthy tissues. The indirect strategies can also be combined with water cooling of the irradiated surface where sensitive pain receptors are nearby the body surface. Indeed, surface cooling will significantly reduce the pain associated with heating a thin superficial layer. Therefore, combining indirect heating of the tumor with surface cooling enables enhanced irradiation doses without unspecific heating and treatment-associated pain [159].

The sensitivity of cancer cells to heat can be increased by suppressing the synthesis of HSPs in these cells to achieve a highly efficient photothermal treatment at lower temperatures. In other words, mild-temperature (≤45 °C) treatment can cause apoptosis in cancer cells if the expression of HSPs is inhibited [160]. In this regard, the HSPs’ cellular synthesis can be inhibited by using specific mesoporous nanoparticles loaded with HSP inhibitors drugs. Sun et al. formulated hollow mesoporous carbon spheres (HMCS) loaded with gambogic acid (GA) for mild photothermal therapy [37]. GA can specifically target and reduce the HSPs in the cytoplasm and then assist in the thermal sensitivity of cells, consequently causing enhanced cell apoptosis at mild temperatures (42–45 °C).

**Figure 7:**
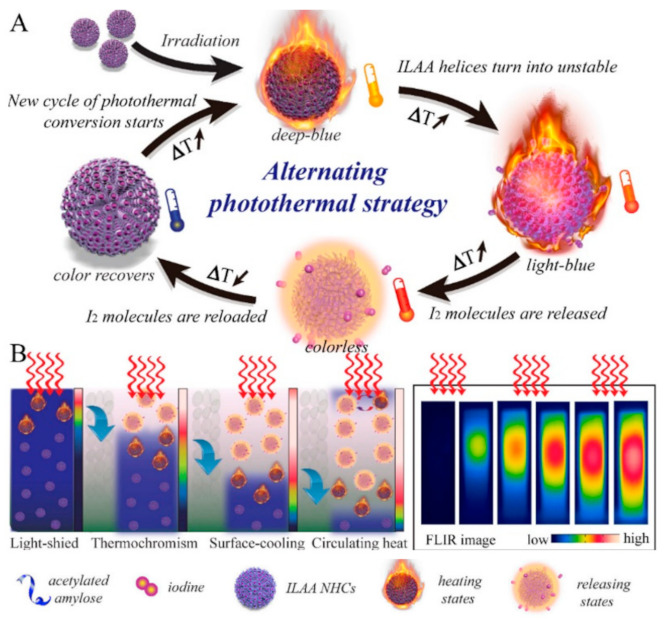
Photothermal strategy based on ILAA NHCs. (**A**) Alternating photothermal cycle based on reversible thermochromic ILAA NHCs for temperature self-regulation. (**B**) The reversible thermochromism-induced enhanced efficient photothermal depth. Optimal ILAA NHCs demonstrate reversible thermochromism in which the color disappears and repeatedly recovers at the proper temperature concerning the I_2_ release and reload on the nanoparticles. Adapted with permission from [57].

Loss of thermo-feedback in PTT with photothermal agents themselves can cause additional adverse effects, such as skin burn, inflammation, and pain in patients. To overcome this issue, a real-time and robust temperature adjustment in the treatment region seems to be necessary [161,162]. Tang et al. designed a thermochromism temperature self-regulation nano-assembly based on iodine (I_2_)-loaded acetylated amylose nano-helix clusters (ILAA NHCs) [57]. When the temperature increased above a critical value during laser irradiation, the helices unfolded, and the I_2_ was gradually released from the ILAA NHCs. At this moment, the ILAA NHCs turned from deep blue to colorless due to I_2_ release and lost their photothermal conversion ability, making the laser penetrate deep into the tissue. Subsequently, the ILAA NHCs’ color recovered by reversible thermochromism due to I_2_ reloading into the ILAA NHCs after decreasing the temperature below the critical value via heat dissipation (Figure 7).

Their results showed that I_2_ efficiently worked as a chemotherapeutic drug and the irradiated region had a lower surface temperature using this versatile nanosystem. In other words, the periodic and repeatable photothermal state effectively decreased the risks associated with continuous heating and managed the local temperature in an acceptable range, inhibiting undesired burns and pain.

**Figure 8:**
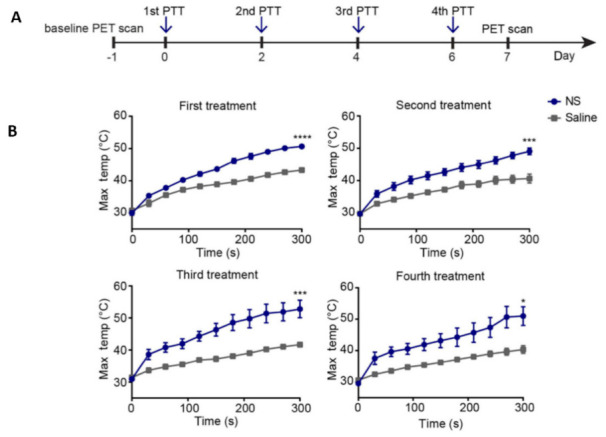
(**A**) The experimental timeline consists of two groups receiving either NS or saline, which were laser-treated four times every other day. (**B**) Temperature increase during all four laser treatments in treatment and the control groups. * *p* < 0.05, *** *p* < 0.001, and **** *p* < 0.0001. Adapted with permission from [166].

Another strategy is to give the treatment in low-dose fractions instead of a single dose, thereby killing cancer cells that survived during the first treatment in following treatments and avoiding surface overheating [163,164,165]. Simón et al. applied a fractionated PTT, using silica–gold nanoshells (NS), instead of one-dose therapy to increase the efficacy of photothermal treatment of subcutaneous colon carcinoma tumors in mice [166]. They evaluated and compared the treatment effect of two different fractionated PTT protocols, using either two or four repeated treatment sessions (Figure 8). 

The mice were kept anesthetized with sevoflurane during the NS injection. The following day, mice were exposed to an NIR laser beam (807 nm, 1.2 W/cm^2^). During the 5 min irradiation, the temperature on the tumor surface was recorded using a real-time FLIR T-440 camera. Their results revealed no significant difference in outcome between groups receiving fractionated PTT compared to a single-dose treatment, despite achieving temperatures above the limit for irreversible damage induction during laser irradiation. Moreover, the survival rate of several mice in fractionated PTT increased compared to single-dose and control animals. The animals were provided pain relief with temgesic (0.3 mg/mL) every 6–8 h for 24 h, starting immediately before the treatment to avoid unnecessary distress and pain.

The intratumoral NP distribution is one of the most crucial parameters in PTT, which can affect the treatment efficiency and outcomes. This parameter is more critical in association with the large tumors that are common in the clinic. Large tumors generally contain hypoxic regions with reduced blood perfusion, and this, as well as high interstitial pressure, prevents nanoparticle delivery to these regions. The uneven NP distribution and the limited laser penetration depth lead to inhomogeneous intratumoral heat distribution during PTT, which decreases the treatment efficiency and makes it challenging to destroy all cancer cells simultaneously [167]. Several approaches for fixing this problem include combining therapies (PTT and immunotherapy), using fractionated PTT explained previously, and fabricating novel nanostructures that improve intratumoral nanoparticle distribution [168,169,170,171]. 

**Figure 9:**
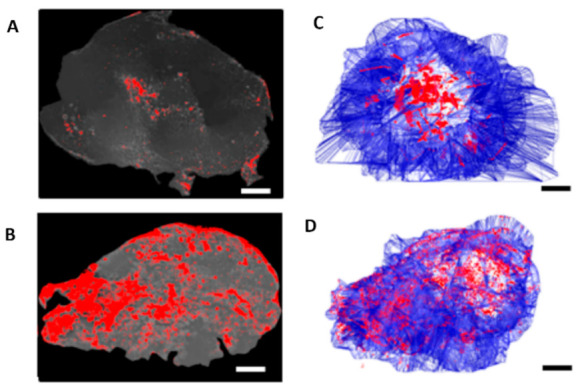
Comparison of free AuNRs’ and NSC-AuNRs’ distribution after intratumoral injection showed that NSCs caused a uniform distribution of AuNRs in the tumor. Three days after AuNR injection, tumors were sectioned and imaged using dark-field microscopy. (**A**,**B**) Mapped cross-sections of tumors injected with free AuNRs (**C**) or NSC-AuNRs (**D**). (**C**,**D**) 3D projection of all mapped AuNRs (red) and tumor (blue) traces generated using reconstruction software in tumors that received free AuNRs (**C**) or NSC-AuNRs (**D**) (Scale bar = 1 mm). Adapted with permission from [172].

Mooney et al. demonstrated that neural stem cells (NSCs) loaded with gold nanorods (AuNRs) can be used to improve the intratumoral distribution of AuNRs and thereby increase the efficacy of PTT (Figure 9). In this research, intratumoral injections of AuNR-loaded NSCs were more effective than free AuNR injection regarding reduced recurrence rates of breast cancer xenografts following PTT [172].

Three days after intratumoral injection of NSC-AuNRs, mice were anesthetized and exposed to NIR laser (811 nm, 2 W/cm^2^) for 5 min. The results showed that the distribution of AuNRs throughout the tumors was improved when transported by NSCs. Buprenex (medication for moderate-to-severe pain relief) was subcutaneously injected into mice once recovered from the anesthesia after laser exposure. 

The pain associated with the PTT procedure might be reduced by an appropriate choice of irradiation parameters such as laser intensity, power, exposure duration, and pattern. In this regard, one preliminary strategy to control tissue overheating is stepwise decreasing the laser power during the treatment or decreasing the laser power along with prolonging the treatment time [117,166]. If high laser powers are used, interstitial laser phototherapy has a high risk of death from extensive tissue carbonization. However, if low powers (in the order of 3 W) are used, a few side effects, such as mild abdominal pain that can radiate to the shoulder or back, can appear [117]. Using painkiller medications as well as nerve blocks during/after treatment seems to be necessary. Besides overheating and catheter insertion, the temperature of the AuNP solution should be checked before injection since cold nanoparticle solutions can cause pain in some patients. 

## 5. Temperature Monitoring during PTT

To control the temperature increase inside the tumor and inhibit the tissue damage due to the overheating, which causes pain during and after treatment, real-time temperature monitoring is necessary for PTT. Besides this, real-time temperature measurement enables the clinician to interrupt the laser exposure in an unpleasant incident such as intolerable pain or burning during the treatment. Using invasive methods for temperature probing during PTT, such as tissue-inserted fiber optics, can be painful by itself and make the patient anxious, hence increasing the pain sensation. The inability to directly monitor the local temperature deep within the tissues is one of the main concerns that face clinical PTT [173]. 

Different temperature measurement methods have been introduced for hyperthermia treatment, including interstitial and intra-luminal thermometry, infrared probing, microwave-based temperature sensing, and magnetic resonance imaging thermometry (MRT) [29,174,175,176,177]. The interstitial and intra-luminal thermometers are inserted in the critical tissues and body cavities, respectively [178]. Despite high accuracy and temporal and spatial resolution, the number of temperature probes and reachable locations are limited. Consequently, invasive thermometry provides only local temperature information, not representing the overall temperature distribution [29,179,180]. 

Furthermore, infrared probes only measure the surface temperature, which is different from the temperature in the tumor and adjacent tissues [174]. Microwave-based temperature sensing penetrates deep inside the body but has a limited spatial resolution due to the long-wavelength radiation used and does not provide high selectivity to chemical subcomponents [175]. MRT relies on temperature-dependent proton resonance frequency shift, which is not always applicable. Other temperature-dependent properties that can be probed using MRI, such as T1 (spin-lattice) and T2 (spin-spin) relaxation time of water protons, are highly complex to calibrate accurately and often costly for many practical applications [181]. Given the importance of monitoring the temperature deep inside the tissue/tumor for reaching an optimum regime (high efficacy/low pain) and associated difficulties in doing so deep inside tissue, developing a non-invasive method for deep temperature monitoring during PTT seems crucial.

Since the optical properties of tissues, such as absorption and scattering, can be affected by their temperature, one can measure the tissue temperature by measuring the change in these properties [182,183,184,185]. In this regard, Raman spectroscopy presents the advantage of enabling temperature measurements with high chemical specificity; however, in its basic form, it is limited to near surfaces within diffusely scattering samples. The principle relies on measuring both the Stokes and anti-Stokes components of the Raman spectrum, and then the local temperature is derived from the ratio of the Stokes and anti-Stokes intensities [186]. The intensity ratio can be approximated by:

(3) Ianti−StokesIStokes=exp−EkT

where E is the first vibrational state energy (J), k is the Boltzmann constant (1.38 × 10^−23^ J K^−1^), and T is the temperature (K).

**Figure 10:**
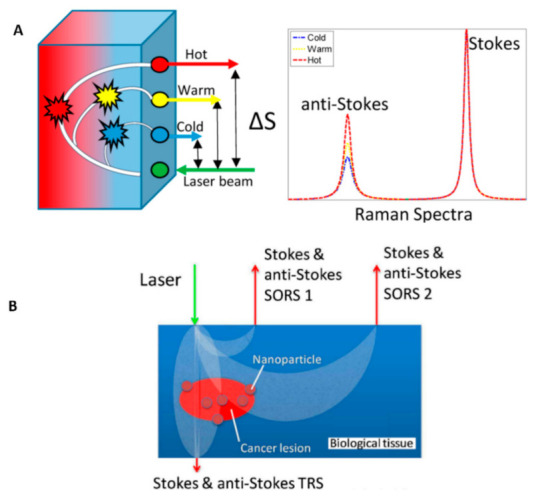
(**A**) Concept of T-SORS showing a cold surface and warm interior; as the spatial offset between the illumination point and collection point increases, there is a relative increase in the anti-Stokes contribution in the spectra originating from the warmer sublayer. (**B**) Schematic diagram of T-SESORS thermometry. The SORS and TRS geometries permit subsurface sample temperature monitoring mediated by SERS nanoparticles embedded in a turbid medium. The temperature of nanoparticles can also be elevated above background levels and monitored using laser radiation. Adapted with permission from [190,191].

To overcome the Raman spectroscopy limitations, spatially offset Raman spectroscopy (SORS) has been introduced with deep penetration inside the turbid media and tissues [187,188,189]. The concept of SORS is that Raman signals collected at a distance Δs on the sample surface from the laser illumination contain larger contributions from the materials buried beneath the surface of the tissue than those collected from the illumination zone [190,191]. Measurement of the Stokes and anti-Stokes parts of the scattering spectrum using SORS leads to a unique method called “Temperature SORS (T-SORS)” (Figure 10).

Gardner et al. made the first demonstration of T-SORS by measuring the temperature of a 3 mm subsurface poly(tetrafluoroethylene) (PTFE) through a 3 mm top layer of poly(oxymethylene) (POM) at near ambient temperature (∼22 °C) [190]. The results showed the possibility of predicting the buried PTFE layer temperature using anti-Stokes/Stokes of PTFE heating (24 to 45 °C). 

Using plasmonic nanoparticles labeled with a Raman reporter enables photothermal ablation and real-time non-invasive temperature monitoring via T-SORS simultaneously. Raman reporter labels adsorbed onto the surface of the noble metal plasmonic nanoparticles cause reporter molecules to have significantly enhanced Raman signals, called surface-enhanced Raman spectroscopy (SERS), enabling their readout from deeper layers of tissue, [192]. In other studies, Gardner et al. demonstrated the simultaneous heating and real-time non-invasive temperature monitoring of subsurface-embedded nanoparticles within turbid materials [191] and biological tissues (T-SESORS) [173]. The latter study even used two different nanoparticles, one for reporting temperature and one for heating (100 nm Au nanospheres and Au nanoshells, respectively), introducing higher laser absorption and increased anti-Stokes/Stokes ratios. 

**Figure 11:**
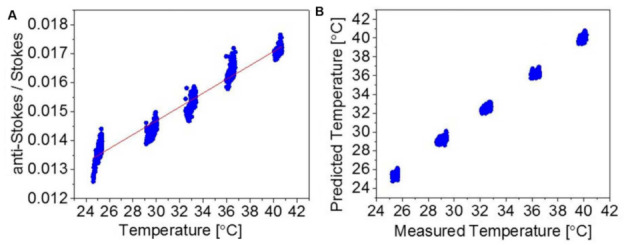
Model of heating AuNPs (25–42 °C) buried in 10 mm porcine tissue. (**A**) The average anti-Stokes/Stokes band of the thiol-based Raman reporter (~1080 cm^−1^) at each temperature point. (**B**) Predicted temperatures vs. the measured temperature of the AuNPs. Adapted with permission from [192].

As mentioned earlier, the T-SESORS approach relies on changing both the anti-Stokes and Stokes signals of molecules attached or adjacent to SERS nanoparticles at different temperatures (Figure 11). 

Overall, T-SESORS can monitor the temperature at depth non-invasively and control the photothermal dose to the treatment zone. Moreover, this temperature monitoring can be achieved in real time to ensure minimum damage to surrounding tissues during photothermal treatment, potentially resulting in more effective pain management. 

**Figure 12:**
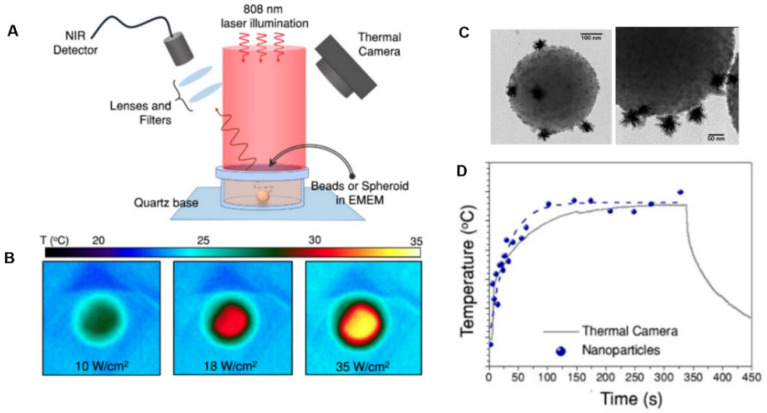
Heating and emitting properties of hybrid beads containing gold nanostars and CaF2:Nd3+, Y3+ nanoparticles. (**A**) Experimental set-up used to illuminate the samples and record their luminescence while monitoring the temperature of the solution with a thermal camera. (**B**) Thermal camera images upon illumination of 1 mL of bead solution with three different powers. (**C**) TEM images of hybrid nanoprobe including bead, gold nanostars, and CaF2:Nd3+, Y3+ nanoparticles. (**D**) Temperature curve of the dispersion of beads over time, illuminated at 2400 mW, measured using a thermal camera (grey squares) and nanothermometry (blue dots). Adapted with permission from [193].

Another method combining heating and temperature monitoring using nanoparticles is using materials capable of heating and emitting a temperature-dependent signal. Quintanilla et al. developed a nanoprobe that allows controlled local heating combined with in situ nanothermometry [193]. This nanoprobe constituted PEGylated gold nanostars (83 ± 7 nm) as photothermal agents along with silica-coated CaF2:Nd3+, Y3+ nanoparticles (11 ± 3 nm) as luminescent nanothermometers mounted on polystyrene beads (500 nm). Gold and CaF2:Nd3+, Y3+ nanoparticles could be simultaneously excited at 808 nm in the first biological window, while the nanothermometer emission was located in the second biological window (1050 nm). In this regard, the luminescence signal from the nanothermometers allowed the tracking and localization of the gold nanostars inside the biological tissue. Hybrid probes were internalized in 3D tumor spheroids to induce cell death via photothermal effects, while simultaneously measuring the local temperature (thermal resolution ~4 °C) in situ (Figure 12).

In CaF2:Nd3+, Y3+ particles, Nd3+ is the active ion absorbing light at 808 nm and featuring a strong emission band at 1050 nm, while Y3+ is added to break energy migration paths between Nd3+ ions, which would otherwise quench the emission intensity [194]. Moreover, lanthanide ions as dopants present characteristic narrow emission bands, which easily differentiate from any other signal from organic molecules in the biological tissues. The lanthanide emission also features a significant Stokes shift, consequently minimizing the absorption of light emitted by nearby gold nanostars. Thermal calibration can be performed by measuring the emission from a colloidal dispersion of the nanoprobes at different temperatures.

The temperature can be measured non-invasively and in real time (time resolution ~30 s) using this method, and by increasing the number of nanothermometers per gold nanostar, the luminescence intensity and time resolution will be improved. In this regard, since gold nanostars have to be slightly (>5 nm) separated from the luminescent nanoparticles to avoid luminescence quenching, there is a limitation in the maximum number of nanothermometers on each polystyrene bead. On the other hand, some other optimizations are needed in this approach, such as reducing the bead size and boosting the heating efficiency of the hybrid nanostructure.

## 6. Conclusions

Since photothermal therapy has proven to be a promising approach for cancer treatment and has shown good results, controlling patients’ pain during treatment is crucial for its effective uptake in clinics. In this review, we discussed different strategies that have been used in the literature to control and manage patient’s pain.

The strategies mentioned in the literature to alleviate the pain mainly for laser interstitial hyperthermia treatment (without nanoparticles) depend on the tumor location and treatment procedure. In hepatic cancer, the pain is mainly in the epigastric region, which can be due to invasive laser and thermocouple fiber insertion; in breast cancer, the pain can be due to overheating the tumor location or skin burning. In these situations, the skin heat can be removed by coolant air or water spray. 

Moreover, any parameter that increases the efficiency of the photothermal treatment can be optimized to manage the patient’s pain. To achieve successful pain management during PTT, we have to consider optimizing as many parameters as possible, including achieving efficient and painless nanoparticle delivery, choosing appropriate laser exposure parameters, and using physical strategies for body cooling during treatment. In this regard, one of the most significant challenges in translating photothermal ablation into the clinic is optimizing the nanoparticles’ delivery. Using an optimal delivery system, patients encounter less pain during treatment, increasing the PTT efficiency. Homogenous distribution of nanoparticles in the tumor guarantees uniform heat in the tissue and enhanced therapeutic outcomes. Moreover, optimizing the involved parameters in the laser–heat conversion efficiency of nanoparticles can achieve higher treatment efficiency and fewer patient complications during and after the PTT procedure.

A set of predetermined and rigid rules cannot be issued and applied for all patients to manage the pain in the clinic, since the pain threshold and tolerance level depend on individual physiological and psychological conditions. Accordingly, each patient’s physiological and psychological states must be considered before starting the treatment course (individualized pain management). Besides this, explaining the treatment procedure for the patients can make them familiar with what they need to go through and decreases their anxiety, which can turn into pseudo-pain during treatment. 

The perception of pain by different patients, or by one patient at different times or at different anatomical locations, depends on several parameters, including their state of mind, previous experiences, and fear and anxiety during PTT. Consequently, any measurement of pain must be taken with caution. In this regard, developing a robust and precise method to measure patients’ pain during the whole treatment course seems necessary. Moreover, monitoring the real-time temperature in PTT is a key parameter inhibiting unwanted tissue damage and consequent pain. To this end, the T-SESORS and nanothermometry are promising approaches, and can pave the way to clinical nanoparticle-based photothermal treatment. 

All in all, better knowledge of the relations between pain, treatment parameters, and clinical outcomes would help physicians to determine the optimal parameters for use in each treatment. As a result, this individualization should enhance the treatment success rate and patients’ comfort. Moreover, a better understanding of the physiological processes involved in pain induced by PTT would help to develop new pain reduction strategies.

## Figures and Tables

**Table 1:** IASP basic pain terminologies.

Terminology	Definition
**Pain**	A subjective unpleasant sensory and emotional experience associated with actual or potential tissue damage.
**Noxious stimulus**	Stimulus that is damaging to normal tissues and causes pain.
**Nociceptive stimulus**	An actually or potentially tissue-damaging event transduced and encoded by nociceptors.
**Nociceptor**	A receptor preferentially sensitive to a noxious stimulus or a prolonged stimulus that would become noxious.
**Pain threshold**	The minimal intensity of a stimulus that is perceived as painful.
**Pain tolerance level**	The maximum intensity of a stimulus that evokes pain and that a subject is able to tolerate in a given situation.

**Table 2:** Photothermal conversion efficiency of different materials.

Materials	Size (nm)	Laser Wavelength (nm)	Laser Irradiance (or Power)	Reported Conversion Efficiency (%)	Ref.
Au nanorods	5 × 27	800	2 W/cm^2^	21	[94]
Au nanorods	10 × 41	808	1 W/cm^2^	17	[95]
Au nanorods	13 × 44	815	-	62	[96]
Au nanorods	15 × 50	980	0.51 W/cm^2^	23.7	[97]
Au nanostars	36	808	2 mW	74	[98]
Au nanoshells	65	800	2 W/cm^2^	13	[94]
Au nanospheres	5	532	228 mW	80	[83]
	15			78	
	19			73	
	40			69.4	
	50			65	
Ag@Ag_2_S core–shell structures	25	635	420 mW	63.7	[99]
		808		64.7	
		1064		79.3	
Au-Ag_2_S hybrid	88 ± 8	809	1.72 W	64	[92]
Au-ZnS hybrid	160 ± 8	809	1.72 W	86	[92]
Au nanopolyhedrons	3 ± 1	809	1.72 W	18	[92]
hollow silicon oxide nanoparticles (H-SiOx NPs)	150	1064	0.6 W/cm^2^	48.6	[100]
copper selenide (Cu2xSe) nanocrystals	16	800	2 W/cm^2^	22	[94]
Cu9S5 nanocrystals	13 ×70	980	0.51 W/cm^2^	25.7	[97]
PEG-coated copper sulfide nanoparticles (CuS)	7.2 ± 0.7	970	0.18 W	71.4	[101]
MoS_2_ nanoflakes	50–300	808	2 W/cm^2^	27	[102]
PEG-coated Ti_2_C nanoflakes	41 ± 8	808	-	87.1	[103]
Ti_2_O_3_ nanoparticles	400	>760	7 kW/cm^2^	82	[104]
hollow Pt nanoframes	30	1120	1.2 W	52.5	[105]
GO	300–700	808	200 mW	4	[106]
		980		2	
carbon-based NPs	100	1064	1.0 W/cm^2^	50.6	[107]
multi-walled carbon nanotubes	10 × 1500	808	1 W/cm^2^	53	[108]
semiconducting polymer nanobioconjugates (SPN)	25	808	1 W/cm^2^	20–30	[95]
polyaniline nanoparticles (PANPs)	48.5 ± 1.5	808	0.5 W/cm^2^	48.5	[109]

**Table 3:** Safety parameters used before, during, and after treatment. Reproduced with permission from [115].

Treatment Stage	Actions
**Before treatment**	Checking possible jaundice, fever, and common cold; complete blood count and coagulation (i.e., hematocrit, prothrombin time, partial thromboplastin time) tests
**During treatment**	Monitoring of heart rate, blood pressure, and blood oxygen level; administration of 1% mepivacaine for local anesthesia, diazepam for mild conscious sedation, single dose of antibiotics (usually, 2 g of cefotiam), and opioids (e.g., piritramide and pethidine) intravenously for pain management
**After treatment** **(immediately)**	Using opioids (e.g., piritramide and pethidine) intravenously for pain, antinausea medication (e.g., metoclopramide), and fluids; continuous monitoring of heart rate; monitoring of blood pressure every 30 min for 6 h
**After treatment** **(delayed)**	Checking possible fever, jaundice, and shortness of breath after ten days

## Data Availability

Not applicable.

## References

[1] Ahmad A.S., Ormiston-Smith N., Sasieni P. (2015). Trends in the lifetime risk of developing cancer in Great Britain: Comparison of risk for those born from 1930 to 1960. Br. J. Cancer.

[2] European Commission (2021). Communication from the Commission to the European Parliament, the Council, the European Economic and Social Committee and the Committee of the Regions on European Missions. European Commission Directorate-General for Research and Innovation Directorate G—Common Policy Centre. https://op.europa.eu/en/publication-detail/-/publication/6a2cfdb8-2101-11ec-bd8e-01aa75ed71a1/language-en.

[3] Ferlay J., Ervik M., Lam F., Colombet M., Mery L., Piñeros M., Znaor A., Soerjomataram I., Bray F. (2020). Global Cancer Observatory: Cancer Tomorrow.

[4] Bao X., Yuan Y., Chen J., Zhang B., Li D., Zhou D., Jing P., Xu G., Wang Y., Hola K. (2018). In vivo theranostics with near-infrared-emitting carbon dots—Highly efficient photothermal therapy based on passive targeting after intravenous administration. Light. Sci. Appl..

[5] Chen Y.-W., Su Y.-L., Hu S.-H., Chen S.-Y. (2016). Functionalized graphene nanocomposites for enhancing photothermal therapy in tumor treatment. Adv. Drug Deliv. Rev..

[6] Cai C., Li X., Wang Y., Liu M., Shi X., Xia J., Shen M. (2019). Polydopamine-coated gold core/hollow mesoporous silica shell particles as a nanoplatform for multimode imaging and photothermal therapy of tumors. Chem. Eng. J..

[7] Lu Y., Wu P., Yin Y., Zhang H., Cai C. (2014). Aptamer-functionalized graphene oxide for highly efficient loading and cancer cell-specific delivery of antitumor drug. J. Mater. Chem. B.

[8] Zhang J., Yuan Z.-F., Wang Y., Chen W.-H., Luo G.-F., Cheng S.-X., Zhuo R.-X., Zhang X.-Z. (2013). Multifunctional Envelope-Type Mesoporous Silica Nanoparticles for Tumor-Triggered Targeting Drug Delivery. J. Am. Chem. Soc..

[9] Ruttala H.B., Chitrapriya N., Kaliraj K., Ramasamy T., Shin W.H., Jeong J.-H., Kim J.R., Ku S.K., Choi H.-G., Yong C.S. (2017). Facile construction of bioreducible crosslinked polypeptide micelles for enhanced cancer combination therapy. Acta Biomater..

[10] Edgar L.J., Vellanki R.N., McKee T.D., Hedley D., Wouters B.G., Nitz M. (2016). Isotopologous Organotellurium Probes Reveal Dynamic Hypoxia In Vivo with Cellular Resolution. Angew. Chem..

[11] Brown S.D., Nativo P., Smith J.-A., Stirling D., Edwards P.R., Venugopal B., Flint D.J., Plumb J.A., Graham D., Wheate N.J. (2010). Gold Nanoparticles for the Improved Anticancer Drug Delivery of the Active Component of Oxaliplatin. J. Am. Chem. Soc..

[12] Deng X., Guan W., Qing X., Yang W., Que Y., Tan L., Liang H., Zhang Z., Wang B., Liu X. (2020). Ultrafast Low-Temperature Photothermal Therapy Activates Autophagy and Recovers Immunity for Efficient Antitumor Treatment. ACS Appl. Mater. Interfaces.

[13] Zhou L., Xi Y., Xue Y., Wang M., Liu Y., Guo Y., Lei B. (2019). Injectable self-healing antibacterial bioactive polypeptide-based hybrid nanosystems for efficiently treating multidrug resistant infection, skin-tumor therapy, and enhancing wound healing. Adv. Funct. Mater..

[14] Chen W.R., Adams R.L., Carubelli R., Nordquist R.E. (1997). Laser-photosensitizer assisted immunotherapy: A novel modality for cancer treatment. Cancer Lett..

[15] Jolesz F.A., Hynynen K. (2002). Magnetic resonance image-guided focused ultrasound surgery. Cancer J..

[16] Gazelle G.S., Goldberg S.N., Solbiati L., Livraghi T. (2000). Tumor Ablation with Radio-frequency Energy. Radiology.

[17] O’Neal D.P., Hirsch L.R., Halas N.J., Payne J.D., West J.L. (2004). Photo-thermal tumor ablation in mice using near infrared-absorbing nanoparticles. Cancer Lett..

[18] Vogel A.A., Venugopalan V. (2003). Mechanisms of Pulsed Laser Ablation of Biological Tissues. Chem. Rev..

[19] Conde J., Oliva-Jorge N., Zhang Y., Artzi N. (2016). Local triple-combination therapy results in tumour regression and prevents recurrence in a colon cancer model. Nat. Mater..

[20] Zeng X., Luo M., Liu G., Wang X., Tao W., Lin Y., Ji X., Nie L., Mei L. (2018). Polydopamine-Modified Black Phosphorous Nanocapsule with Enhanced Stability and Photothermal Performance for Tumor Multimodal Treatments. Adv. Sci..

[21] Wu P., Deng D., Gao J., Cai C. (2016). Tubelike Gold Sphere–Attapulgite Nanocomposites with a High Photothermal Conversion Ability in the Near-Infrared Region for Enhanced Cancer Photothermal Therapy. ACS Appl. Mater. Interfaces.

[22] Qin Z., Bischof J.C. (2012). Thermophysical and biological responses of gold nanoparticle laser heating. Chem. Soc. Rev..

[23] Yang X., Yang M., Pang B., Vara M., Xia Y. (2015). Gold Nanomaterials at Work in Biomedicine. Chem. Rev..

[24] Lin T.-C., Lin F.-H., Lin J.-C. (2012). In vitro feasibility study of the use of a magnetic electrospun chitosan nanofiber composite for hyperthermia treatment of tumor cells. Acta Biomater..

[25] Bettaieb A., Wrzal P.K., Averill-Bates D.A. (2013). Hyperthermia: Cancer treatment and beyond. Cancer treatment-conventional and innovative approaches. Cancer Treatment: Conventional and Innovative Approaches.

[26] Takada T., Yamashita T., Sato M., Sato A., Ono I., Tamura Y., Sato N., Miyamoto A., Ito A., Honda H. (2009). Growth Inhibition of Re-Challenge B16 Melanoma Transplant by Conjugates of Melanogenesis Substrate and Magnetite Nanoparticles as the Basis for Developing Melanoma-Targeted Chemo-Thermo-Immunotherapy. J. Biomed. Biotechnol..

[27] Babbs C.F., DeWitt D.P. (1981). Physical principles of local heat therapy for cancer. Med. Instrum..

[28] Sugahara T., Van Der Zee J., Kampinga H.H., Vujaskovic Z., Kondo M., Ohnishi T., Li G., Park H.J., Leeper D.B., Ostapenko V. (2008). Kadota Fund International Forum 2004. Application of thermal stress for the improvement of health, 15–18 June 2004, Awaji Yumebutai International Conference Center, Awaji Island, Hyogo, Japan. Final Report. Int. J. Hyperth..

[29] Van Rhoon G.C., Wust P. (2005). Introduction: Non-invasive thermometry for thermotherapy. Int. J. Hyperth..

[30] Cihoric N., Tsikkinis A., van Rhoon G., Crezee H., Aebersold D.M., Bodis S., Beck M., Nadobny J., Budach V., Wust P. (2015). Hyperthermia-related clinical trials on cancer treatment within the ClinicalTrials. gov registry. Int. J. Hyperth..

[31] Fan W., Yung B., Huang P., Chen X. (2017). Nanotechnology for Multimodal Synergistic Cancer Therapy. Chem. Rev..

[32] Weissleder R. (2001). A clearer vision for in vivo imaging. Nat. Biotechnol..

[33] Antaris A.L., Robinson J.T., Yaghi O.K., Hong G., Diao S., Luong R., Dai H. (2013). Ultra-Low Doses of Chirality Sorted (6,5) Carbon Nanotubes for Simultaneous Tumor Imaging and Photothermal Therapy. ACS Nano.

[34] Huang X., Jain P.K., El-Sayed I.H., El-Sayed M.A. (2007). Gold nanoparticles: Interesting optical properties and recent applications in cancer diagnostics and therapy. Nanomedicine.

[35] Chen Q., Qi H., Ruan L., Ren Y. (2017). Experimental Comparison of Photothermal Conversion Efficiency of Gold Nanotriangle and Nanorod in Laser Induced Thermal Therapy. Nanomaterials.

[36] Deng H., Zhong Y., Du M., Liu Q., Fan Z., Dai F., Zhang X. (2014). Theranostic Self-Assembly Structure of Gold Nanoparticles for NIR Photothermal Therapy and X-Ray Computed Tomography Imaging. Theranostics.

[37] Sun J., Li Y., Teng Y., Wang S., Guo J., Wang C. (2020). NIR-controlled HSP90 inhibitor release from hollow mesoporous nanocarbon for synergistic tumor photothermal therapy guided by photoacoustic imaging. Nanoscale.

[38] Zhu X., Feng W., Chang J., Tan Y.-W., Li J., Chen M., Sun Y., Li F. (2016). Temperature-feedback upconversion nanocomposite for accurate photothermal therapy at facile temperature. Nat. Commun..

[39] Huang P., Lin J., Li W., Rong P., Wang Z., Wang S., Wang X., Sun X., Aronova M., Niu G. (2013). Biodegradable Gold Nanovesicles with an Ultrastrong Plasmonic Coupling Effect for Photoacoustic Imaging and Photothermal Therapy. Angew. Chem..

[40] Chen W., Qin M., Chen X., Wang Q., Zhang Z., Sun X. (2018). Combining photothermal therapy and immunotherapy against melanoma by polydopamine-coated Al_2_O_3_ nanoparticles. Theranostics.

[41] Marangon I., Ménard-Moyon C., Silva A.K., Bianco A., Luciani N., Gazeau F. (2016). Synergic mechanisms of photothermal and photodynamic therapies mediated by photosensitizer/carbon nanotube complexes. Carbon.

[42] Moon H.K., Lee S.H., Choi H.C. (2009). In Vivo Near-Infrared Mediated Tumor Destruction by Photothermal Effect of Carbon Nanotubes. ACS Nano.

[43] Yang K., Zhang S., Zhang G., Sun X., Lee S.-T., Liu Z. (2010). Graphene in Mice: Ultrahigh In Vivo Tumor Uptake and Efficient Photothermal Therapy. Nano Lett..

[44] Yang K., Hu L., Ma X., Ye S., Cheng L., Shi X., Li C., Li Y., Liu Z. (2012). Multimodal Imaging Guided Photothermal Therapy using Functionalized Graphene Nanosheets Anchored with Magnetic Nanoparticles. Adv. Mater..

[45] Thakur M., Kumawat M.K., Srivastava R. (2017). Multifunctional graphene quantum dots for combined photothermal and photodynamic therapy coupled with cancer cell tracking applications. RSC Adv..

[46] Jaque D., Maestro L.M., del Rosal B., Haro-Gonzalez P., Benayas A., Plaza J.L., Rodríguez E.M., Solé J. (2014). Nanoparticles for photothermal therapies. Nanoscale.

[47] Aqel A., Abou El-Nour K.M.M., Ammar R.A.A., Al-Warthan A. (2012). Carbon nanotubes, science and technology part (I) structure, synthesis and characterisation. Arab. J. Chem..

[48] Zhang B., Wang Y., Zhai G. (2016). Biomedical applications of the graphene-based materials. Mater. Sci. Eng. C.

[49] Miao W., Shim G., Lee S., Oh Y.-K. (2014). Structure-dependent photothermal anticancer effects of carbon-based photoresponsive nanomaterials. Biomaterials.

[50] Shirshahi V., Hatamie S., Tabatabaei S.N., Salimi M., Saber R. (2018). Enhanced Thermal Stability and Biocompatibility of Gold Nanorods by Graphene Oxide. Plasmonics.

[51] Terentyuk G.S., Maslyakova G., Suleymanova L.V., Khlebtsov N., Khlebtsov B., Akchurin G.G., Maksimova I.L., Tuchin V. (2009). Laser-induced tissue hyperthermia mediated by gold nanoparticles: Toward cancer phototherapy. J. Biomed. Opt..

[52] Zhang Y., Xiu W., Sun Y., Zhu D., Zhang Q., Yuwen L., Weng L., Teng Z., Wang L. (2017). RGD-QD-MoS2 nanosheets for targeted fluorescent imaging and photothermal therapy of cancer. Nanoscale.

[53] Wang Z., Ma Y., Yu X., Niu Q., Han Z., Wang H., Li T., Fu D., Achilefu S., Qian Z. (2018). Targeting CXCR4–CXCL12 axis for visualizing, predicting, and inhibiting breast cancer metastasis with theranostic AMD3100–Ag2S quantum dot probe. Adv. Funct. Mater..

[54] Zhao H., Li L., Zhang J., Zheng C., Ding K., Xiao H., Wang L., Zhang Z. (2018). C–C Chemokine Ligand 2 (CCL2) Recruits Macrophage-Membrane-Camouflaged Hollow Bismuth Selenide Nanoparticles to Facilitate Photothermal Sensitivity and Inhibit Lung Metastasis of Breast Cancer. ACS Appl. Mater. Interfaces.

[55] Cao H., Zou L., He B., Zeng L., Huang Y., Yu H., Zhang P., Yin Q., Zhang Z., Li Y. (2017). Albumin Biomimetic Nanocorona Improves Tumor Targeting and Penetration for Synergistic Therapy of Metastatic Breast Cancer. Adv. Funct. Mater..

[56] Su J., Sun H., Meng Q., Yin Q., Zhang P., Zhang Z., Yu H., Li Y. (2016). Bioinspired Nanoparticles with NIR-Controlled Drug Release for Synergetic Chemophotothermal Therapy of Metastatic Breast Cancer. Adv. Funct. Mater..

[57] Tang P., Liu Y., Liu Y., Meng H., Liu Z., Li K., Wu D. (2019). Thermochromism-induced temperature self-regulation and alternating photothermal nanohelix clusters for synergistic tumor chemo/photothermal therapy. Biomaterials.

[58] El-Sayed I.H., Huang X., El-Sayed M.A. (2006). Selective laser photo-thermal therapy of epithelial carcinoma using anti-EGFR antibody conjugated gold nanoparticles. Cancer Lett..

[59] Zhang C., Yong Y., Song L., Dong X., Zhang X., Liu X., Gu Z., Zhao Y., Hu Z. (2016). Multifunctional WS2@ Poly (ethylene imine) Nanoplatforms for Imaging Guided Gene-Photothermal Synergistic Therapy of Cancer. Adv. Healthc. Mater..

[60] Pan L., Liu J., Shi J. (2017). Nuclear-Targeting Gold Nanorods for Extremely Low NIR Activated Photothermal Therapy. ACS Appl. Mater. Interfaces.

[61] Treede R.-D. (2018). The International Association for the Study of Pain definition of pain: As valid in 2018 as in 1979, but in need of regularly updated footnotes. Pain Rep..

[62] Merskey H. (1979). Pain terms: A list with definitions and notes on usage. Recommended by the IASP Subcommittee on Taxonomy. Pain.

[63] Raja S.N., Carr D.B., Cohen M., Finnerup N.B., Flor H., Gibson S., Keefe F.J., Mogil J.S., Ringkamp M., Sluka K.A. (2020). The revised International Association for the Study of Pain definition of pain: Concepts, challenges, and compromises. Pain.

[64] Anand K.J., Craig K.D. (1996). New perspectives on the definition of pain. Pain.

[65] Williams A.C.d.C., Craig K.D. (2016). Updating the definition of pain. Pain.

[66] Cohen M., Quintner J., Van Rysewyk S. (2018). Reconsidering the International Association for the Study of Pain definition of pain. Pain Rep..

[67] Aydede M. (2019). Does the IASP definition of pain need updating?. Pain Rep..

[68] Wells J., Kao C., Mariappan K.G., Albea J., Jansen E.D., Konrad P., Mahadevan-Jansen A. (2005). Optical stimulation of neural tissue in vivo. Opt. Lett..

[69] Wells J., Kao C., Jansen E.D., Konrad P., Mahadevan-Jansen A. (2005). Application of infrared light for in vivo neural stimulation. J. Biomed. Opt..

[70] Loeser J.D., Treede R.-D. (2008). The Kyoto protocol of IASP basic pain Terminology. Pain.

[71] Argoff C.E., Dubin A., Pilitsis J., McCleane G. (2009). Pain Management Secrets E-Book.

[72] Von Ungern-Sternberg B.S., Habre W. (2007). Pediatric anesthesia—Potential risks and their assessment: Part I. Pediatric Anesth..

[73] UnicStojanovic D., Babic S., Jovic M. (2012). Benefits, Risks and Complications of Perioperative Use of Epidural Anesthesia. Med. Arch..

[74] Wernli K.J., Brenner A., Rutter C.M., Inadomi J. (2016). Risks Associated with Anesthesia Services During Colonoscopy. Gastroenterology.

[75] Maestro L.M., Camarillo E., Sánchez-Gil J.A., Rodríguez-Oliveros R., Ramiro-Bargueño J., Caamaño A.J., Jaque F., Solé J.G., Jaque D. (2014). Gold nanorods for optimized photothermal therapy: The influence of irradiating in the first and second biological windows. RSC Adv..

[76] Riley R.S., Day E.S. (2017). Gold nanoparticle-mediated photothermal therapy: Applications and opportunities for multimodal cancer treatment. WIREs Nanomed. Nanobiotechnol..

[77] Loo C., Lin A., Hirsch L., Lee M.-H., Barton J., Halas N., West J., Drezek R. (2004). Nanoshell-Enabled Photonics-Based Imaging and Therapy of Cancer. Technol. Cancer Res. Treat..

[78] Bickford L., Sun J., Fu K., Lewinski N., Nammalvar V., Chang J., Drezek R. (2008). Enhanced multi-spectral imaging of live breast cancer cells using immunotargeted gold nanoshells and two-photon excitation microscopy. Nanotechnology.

[79] Hwang S., Nam J., Jung S., Song J., Doh H., Kim S. (2014). Gold nanoparticle-mediated photothermal therapy: Current status and future perspective. Nanomedicine.

[80] Melamed J., Edelstein R.S., Day E. (2015). Elucidating the Fundamental Mechanisms of Cell Death Triggered by Photothermal Therapy. ACS Nano.

[81] Pérez-Hernández M., Del Pino P., Mitchell S.G., Moros M., Stepien G., Pelaz B., Parak W.J., Gálvez E.M., Pardo J., De La Fuente J.M. (2015). Dissecting the Molecular Mechanism of Apoptosis during Photothermal Therapy Using Gold Nanoprisms. ACS Nano.

[82] Lee J., Chatterjee D.K., Lee M.H., Krishnan S. (2014). Gold nanoparticles in breast cancer treatment: Promise and potential pitfalls. Cancer Lett..

[83] Jiang K., Smith D.A., Pinchuk A. (2013). Size-Dependent Photothermal Conversion Efficiencies of Plasmonically Heated Gold Nanoparticles. J. Phys. Chem. C.

[84] Miyako E., Kono K., Yuba E., Hosokawa C., Nagai H., Hagihara Y. (2012). Carbon nanotube–liposome supramolecular nanotrains for intelligent molecular-transport systems. Nat. Commun..

[85] Baffou G., Quidant R. (2013). Thermo-plasmonics: Using metallic nanostructures as nano-sources of heat. Laser Photon-Rev..

[86] Ansari M.A., Erfanzadeh M., Mohajerani E. (2013). Mechanisms of Laser-Tissue Interaction: II. Tissue Thermal Properties. J. Lasers Med. Sci..

[87] Almada M., Leal-Martínez B., Hassan N., Kogan M., Burboa M., Topete A., Valdez M., Juárez J. (2017). Photothermal conversion efficiency and cytotoxic effect of gold nanorods stabilized with chitosan, alginate and poly(vinyl alcohol). Mater. Sci. Eng. C.

[88] Roper D.K., Ahn W., Hoepfner M. (2007). Microscale Heat Transfer Transduced by Surface Plasmon Resonant Gold Nanoparticles. J. Phys. Chem. C.

[89] Rashidi-Huyeh M., Palpant B. (2004). Thermal response of nanocomposite materials under pulsed laser excitation. J. Appl. Phys..

[90] Wilson O.M., Hu X., Cahill D.G., Braun P.V. (2002). Colloidal metal particles as probes of nanoscale thermal transport in fluids. Phys. Rev. B.

[91] Plech A., Kotaidis V., Grésillon S., Dahmen C., von Plessen G. (2004). Laser-induced heating and melting of gold nanoparticles studied by time-resolved x-ray scattering. Phys. Rev. B.

[92] Chen H., Shao L., Ming T., Sun Z., Zhao C., Yang B., Wang J. (2010). Understanding the Photothermal Conversion Efficiency of Gold Nanocrystals. Small.

[93] Li J., Zhang W., Ji W., Wang J., Wang N., Wu W., Wu Q., Hou X., Hu W., Li L. (2021). Near infrared photothermal conversion materials: Mechanism, preparation, and photothermal cancer therapy applications. J. Mater. Chem. B.

[94] Hessel C.M., Pattani V.P., Rasch M., Panthani M.G., Koo B., Tunnell J.W., Korgel B.A. (2011). Copper Selenide Nanocrystals for Photothermal Therapy. Nano Lett..

[95] Lyu Y., Xie C., Chechetka S.A., Miyako E., Pu K. (2016). Semiconducting Polymer Nanobioconjugates for Targeted Photothermal Activation of Neurons. J. Am. Chem. Soc..

[96] Cole J.R., Mirin N.A., Knight M.W., Goodrich G.P., Halas N.J. (2009). Photothermal Efficiencies of Nanoshells and Nanorods for Clinical Therapeutic Applications. J. Phys. Chem. C.

[97] Tian Q., Jiang F., Zou R., Liu Q., Chen Z., Zhu M., Yang S., Wang J., Wang J., Hu J. (2011). Hydrophilic Cu9S5 Nanocrystals: A Photothermal Agent with a 25.7% Heat Conversion Efficiency for Photothermal Ablation of Cancer Cells in Vivo. ACS Nano.

[98] Maestro L.M., Gonzalez P.H., Sánchez-Iglesias A., Liz-Marzán L.M., Solé J.G., Jaque D. (2014). Quantum Dot Thermometry Evaluation of Geometry Dependent Heating Efficiency in Gold Nanoparticles. Langmuir.

[99] Jiang Q., Zeng W., Zhang C., Meng Z., Wu J., Zhu Q., Wu D., Zhu H. (2017). Broadband absorption and enhanced photothermal conversion property of octopod-like Ag@Ag2S core@shell structures with gradually varying shell thickness. Sci. Rep..

[100] Yu X., Yang K., Chen X., Li W. (2017). Black hollow silicon oxide nanoparticles as highly efficient photothermal agents in the second near-infrared window for in vivo cancer therapy. Biomaterials.

[101] Marin R., Skripka A., Besteiro L.V., Benayas A., Wang Z., Govorov A.O., Canton P., Vetrone F. (2018). Highly Efficient Copper Sulfide-Based Near-Infrared Photothermal Agents: Exploring the Limits of Macroscopic Heat Conversion. Small.

[102] Feng W., Chen L., Qin M., Zhou X., Zhang Q., Miao Y., Qiu K., Zhang Y., He C. (2015). Flower-like PEGylated MoS2 nanoflakes for near-infrared photothermal cancer therapy. Sci. Rep..

[103] Szuplewska A., Kulpińska D., Dybko A., Jastrzębska A.M., Wojciechowski T., Rozmysłowska A., Chudy M., Grabowska-Jadach I., Ziemkowska W., Brzózka Z. (2019). 2D Ti2C (MXene) as a novel highly efficient and selective agent for photothermal therapy. Mater. Sci. Eng. C.

[104] Wang J., Li Y., Deng L., Wei N., Weng Y., Dong S., Qi D., Qiu J., Chen X., Wu T. (2016). High-Performance Photothermal Conversion of Narrow-Bandgap Ti_2_O_3_ Nanoparticles. Adv. Mater..

[105] Wang Q., Wang H., Yang Y., Jin L., Liu Y., Wang Y., Yan X., Xu J., Gao R., Lei P. (2019). Plasmonic Pt Superstructures with Boosted Near-Infrared Absorption and Photothermal Conversion Efficiency in the Second Biowindow for Cancer Therapy. Adv. Mater..

[106] Savchuk O.A., Carvajal J.J., Massons J., Aguiló M., Díaz F. (2016). Determination of photothermal conversion efficiency of graphene and graphene oxide through an integrating sphere method. Carbon.

[107] Guan Q., Zhou L.-L., Zhou L.-N., Li M., Qin G.-X., Li W.-Y., Li Y.-A., Dong Y.-B. (2020). A carbon nanomaterial derived from a nanoscale covalent organic framework for photothermal therapy in the NIR-II biowindow. Chem. Commun..

[108] Maestro L.M., Haro-González P., del Rosal B., Ramiro J., Caamaño A.J., Carrasco E., Juarranz A., Sanz-Rodríguez F., Solé J.G., Jaque D. (2013). Heating efficiency of multi-walled carbon nanotubes in the first and second biological windows. Nanoscale.

[109] Zhou J., Lu Z., Zhu X., Wang X., Liao Y., Ma Z., Li F. (2013). NIR photothermal therapy using polyaniline nanoparticles. Biomaterials.

[110] Liu Z., Cheng L., Zhang L., Yang Z., Liu Z., Fang J. (2014). Sub-100 nm hollow Au–Ag alloy urchin-shaped nanostructure with ultrahigh density of nanotips for photothermal cancer therapy. Biomaterials.

[111] Sebastian V., Lee S.-K., Jensen K.F. (2014). Engineering the synthesis of silica–gold nano-urchin particles using continuous synthesis. Nanoscale.

[112] Nolsøe C.P., Torp-Pedersen S., Burcharth F., Horn T., Christensen N.E., Olldag E.S., Andersen P.H., Karstrup S., Lorentzen T. (1993). Interstitial hyperthermia of colorectal liver metastases with a US-guided Nd-YAG laser with a diffuser tip: A pilot clinical study. Radiology.

[113] Amin Z., Donald J.J., Masters A., Kant R., Steger A.C., Bown S.G., Lees W.R. (1993). Hepatic metastases: Interstitial laser photocoagulation with real-time US monitoring and dynamic CT evaluation of treatment. Radiology.

[114] Masters A., Steger A., Lees W., Walmsley K., Bown S. (1992). Interstitial laser hyperthermia: A new approach for treating liver metastases. Br. J. Cancer.

[115] Vogl T.J., Straub R., Eichler K., Woitaschek D., Mack M.G. (2002). Malignant Liver Tumors Treated with MR Imaging–guided Laser-induced Thermotherapy: Experience with Complications in 899 Patients (2520 lesions). Radiology.

[116] Harries S.A., Amin Z., Smith M.E.F., Lees W.R., Cooke J., Cook M.G., Scurr J.H., Kissin M.W., Bown S.G. (1994). Interstitial laser photocoagulation as a treatment for breast cancer. Br. J. Surg..

[117] Robinson D.S., Parel Ing J.-M., Denham D.B., González-Cirre X., Schachner R.D., Herron A.J., Comander J., Hauptmann G. (1998). Interstitial laser hyperthermia model development for minimally invasive therapy of breast carcinoma. J. Am. Coll. Surg..

[118] Dowlatshahi K., Francescatti D.S., Bloom K.J. (2002). Laser therapy for small breast cancers. Am. J. Surg..

[119] Rastinehad A.R., Anastos H., Wajswol E., Winoker J.S., Sfakianos J.P., Doppalapudi S.K., Carrick M.R., Knauer C.J., Taouli B., Lewis S.C. (2019). Gold nanoshell-localized photothermal ablation of prostate tumors in a clinical pilot device study. Proc. Natl. Acad. Sci. USA.

[120] Staves B. (2010). Pilot Study of AurolaseTM Therapy in Refractory and/or Recurrent Tumors of the Head and Neck. ClinicalTrials. gov Identifier: NCT00848042. NCT00848042.

[121] Valentine R.M., Ibbotson S.H., Brown C.T.A., Wood K., Moseley H. (2011). A quantitative comparison of 5-aminolaevulinic acid-and methyl aminolevulinate-induced fluorescence, photobleaching and pain during photodynamic therapy. Photochem. Photobiol..

[122] Ang J.M., bin Riaz I., Kamal M.U., Paragh G., Zeitouni N.C. (2017). Photodynamic therapy and pain: A systematic review. Photodiagnosis Photodyn. Ther..

[123] Fink C., Enk A., Gholam P. (2015). Photodynamic therapy–aspects of pain management. J. Dtsch. Dermatol. Ges..

[124] Haimi-Cohen R., Cohen A., Carmon A. (1983). A model for the temperature distribution in skin noxiously stimulated by a brief pulse of CO_2_ laser radiation. J. Neurosci. Methods.

[125] Spiegel J., Hansen C., Treede R.-D. (2000). Clinical evaluation criteria for the assessment of impaired pain sensitivity by thulium-laser evoked potentials. Clin. Neurophysiol..

[126] Arits A., Van De Weert M., Nelemans P., Kelleners-Smeets N. (2010). Pain during topical photodynamic therapy: Uncomfortable and unpredictable. J. Eur. Acad. Dermatol. Venereol..

[127] Borelli C., Herzinger T., Merk K., Berking C., Kunte C., Plewig G., Degitz K. (2007). Effect of Subcutaneous Infiltration Anesthesia on Pain in Photodynamic Therapy: A Controlled Open Pilot Trial. Dermatol. Surg..

[128] Tyrrell J., Campbell S., Curnow A. (2011). The effect of air cooling pain relief on protoporphyrin IX photobleaching and clinical efficacy during dermatological photodynamic therapy. J. Photochem. Photobiol. B Biol..

[129] Apalla Z., Sotiriou E., Panagiotidou D., Lefaki I., Goussi C., Ioannides D. (2011). The impact of different fluence rates on pain and clinical outcome in patients with actinic keratoses treated with photodynamic therapy. Photodermatol. Photoimmunol. Photomed..

[130] Babilas P., Knobler R., Hummel S., Gottschaller C., Maisch T., Koller M., Landthaler M., Szeimies R.-M. (2007). Variable pulsed light is less painful than light-emitting diodes for topical photodynamic therapy of actinic keratosis: A prospective randomized controlled trial. Br. J. Dermatol..

[131] Stangeland K., Kroon S. (2012). Cold air analgesia as pain reduction during photodynamic therapy of actinic keratoses. J. Eur. Acad. Dermatol. Venereol..

[132] Chaves Y.N., Torezan L.A., Niwa A.B.M., Sanches A.S., Neto C.F. (2012). Pain in photodynamic therapy: Mechanism of action and management strategies. Anais Bras. Dermatol..

[133] Halldin C., Paoli J., Sandberg C., Gonzalez H., Wennberg A.-M. (2009). Nerve blocks enable adequate pain relief during topical photodynamic therapy of field cancerization on the forehead and scalp. Br. J. Dermatol..

[134] Serra-Guillén C., Hueso L., Nagore E., Vila M., Llombart B., Caballero C.R., Botella-Estrada R., Sanmartín O., Alfaro-Rubio A. (2009). Comparative study between cold air analgesia and supraorbital and supratrochlear nerve block for the management of pain during photodynamic therapy for actinic keratoses of the frontotemporal zone. Br. J. Dermatol..

[135] Lumpkin E.A., Caterina M.J. (2007). Mechanisms of sensory transduction in the skin. Nature.

[136] Beissner F., Brandau A., Henke C., Felden L., Baumgärtner U., Treede R.-D., Oertel B.G., Lotsch J. (2010). Quick Discrimination of Adelta and C Fiber Mediated Pain Based on Three Verbal Descriptors. PLoS ONE.

[137] Li X., Naylor M.F., Le H., Nordquist R.E., Teague T.K., Howard C.A., Murray C., Chen W.R. (2010). Clinical effects of in situ photoimmunotherapy on late-stage melanoma patients: A preliminary study. Cancer Biol. Ther..

[138] Barge J., Glanzmann T., Zellweger M., Salomon D., Bergh H.V.D., Wagnières G. (2013). Correlations between photoactivable porphyrins’ fluorescence, erythema and the pain induced by PDT on normal skin using ALA-derivatives. Photodiagn. Photodyn. Ther..

[139] Johnson C. (2005). Measuring pain. Visual analog scale versus numeric pain scale: What is the difference?. J. Chiropr. Med..

[140] Langley G.B., Sheppeard H. (1985). The visual analogue scale: Its use in pain measurement. Rheumatol. Int..

[141] Duncan K.J. Laser based power transmission: Component selection and laser hazard analysis. Proceedings of the 2016 IEEE PELS Workshop on Emerging Technologies: Wireless Power Transfer (WoW).

[142] Jung B.-K., Lee Y.K., Hong J., Ghandehari H., Yun C.-O. (2016). Mild Hyperthermia Induced by Gold Nanorod-Mediated Plasmonic Photothermal Therapy Enhances Transduction and Replication of Oncolytic Adenoviral Gene Delivery. ACS Nano.

[143] Kaneti Y.V., Chen C., Liu M., Wang X., Yang J.L., Taylor R.A., Jiang X., Yu A. (2015). Carbon-Coated Gold Nanorods: A Facile Route to Biocompatible Materials for Photothermal Applications. ACS Appl. Mater. Interfaces.

[144] Yang Y., Zhu W., Dong Z., Chao Y., Xu L., Chen M., Liu Z. (2017). 1D Coordination Polymer Nanofibers for Low-Temperature Photothermal Therapy. Adv. Mater..

[145] Zhang Y., Sha R., Zhang L., Zhang W., Jin P., Xu W., Ding J., Lin J., Qian J., Yao G. (2018). Harnessing copper-palladium alloy tetrapod nanoparticle-induced pro-survival autophagy for optimized photothermal therapy of drug-resistant cancer. Nat. Commun..

[146] Ren X., Chen Y., Peng H., Fang X., Zhang X., Chen Q., Wang X., Yang W., Sha X. (2018). Blocking Autophagic Flux Enhances Iron Oxide Nanoparticle Photothermal Therapeutic Efficiency in Cancer Treatment. ACS Appl. Mater. Interfaces.

[147] Tian G., Zhang X., Zheng X., Yin W., Ruan L., Liu X., Zhou L., Yan L., Li S., Gu Z. (2014). Multifunctional RbxWO3 Nanorods for Simultaneous Combined Chemo-photothermal Therapy and Photoacoustic/CT Imaging. Small.

[148] Li W., Peng J., Tan L., Wu J., Shi K., Qu Y., Wei X., Qian Z. (2016). Mild photothermal therapy/photodynamic therapy/chemotherapy of breast cancer by Lyp-1 modified Docetaxel/IR820 Co-loaded micelles. Biomaterials.

[149] Sarkar S., Korolchuk V.I., Renna M., Imarisio S., Fleming A., Williams A., Garcia-Arencibia M., Rose C., Luo S., Underwood B.R. (2011). Complex inhibitory effects of nitric oxide on autophagy. Mol. Cell.

[150] Zhang X., Guo Z., Liu J., Tian G., Chen K., Yu S., Gu Z. (2017). Near infrared light triggered nitric oxide releasing platform based on upconversion nanoparticles for synergistic therapy of cancer stem-like cells. Sci. Bull..

[151] Zhang X., Du J., Guo Z., Yu J., Gao Q., Yin W., Zhu S., Gu Z., Zhao Y. (2019). Efficient Near Infrared Light Triggered Nitric Oxide Release Nanocomposites for Sensitizing Mild Photothermal Therapy. Adv. Sci..

[152] Wang J., Liu J., Liu Y., Wang L., Cao M., Ji Y., Wu X., Xu Y., Bai B., Miao Q. (2016). Gd-Hybridized Plasmonic Au-Nanocomposites Enhanced Tumor-Interior Drug Permeability in Multimodal Imaging-Guided Therapy. Adv. Mater..

[153] Wei S., Quan G., Lu C., Pan X., Wu C. (2020). Dissolving microneedles integrated with pH-responsive micelles containing AIEgen with ultra-photostability for enhancing melanoma photothermal therapy. Biomater. Sci..

[154] Ma G., Wu C. (2017). Microneedle, bio-microneedle and bio-inspired microneedle: A review. J. Control. Release.

[155] Kaushik S., Hord A.H., Denson D.D., McAllister D.V., Smitra S., Allen M.G., Prausnitz M.R. (2001). Lack of pain associated with microfabricated microneedles. Anesth. Analg..

[156] Arya J., Henry S., Kalluri H., McAllister D.V., Pewin W.P., Prausnitz M.R. (2017). Tolerability, usability and acceptability of dissolving microneedle patch administration in human subjects. Biomaterials.

[157] Landry J., Chrétien P., Bernier D., Nicole L.M., Marceau N., Tanguay R.M. (1982). Thermotolerance and heat shock proteins induced by hyperthermia in rat liver cells. Int. J. Radiat. Oncol..

[158] Dombrovsky L.A., Timchenko V., Jackson M., Yeoh G.H. (2011). A combined transient thermal model for laser hyperthermia of tumors with embedded gold nanoshells. Int. J. Heat Mass Transf..

[159] Dombrovsky L.A., Timchenko V., Jackson M. (2012). Indirect heating strategy for laser induced hyperthermia: An advanced thermal model. Int. J. Heat Mass Transf..

[160] Davenport J., Manjarrez J.R., Peterson L., Krumm B., Blagg B.S.J., Matts R.L. (2011). Gambogic Acid, a Natural Product Inhibitor of Hsp90. J. Nat. Prod..

[161] Ju Y., Zhang H., Yu J., Tong S., Tian N., Wang Z., Wang X., Su X., Chu X., Lin J. (2017). Monodisperse Au–Fe_2_C Janus Nanoparticles: An Attractive Multifunctional Material for Triple-Modal Imaging-Guided Tumor Photothermal Therapy. ACS Nano.

[162] Lin H., Gao S., Dai C., Chen Y., Shi J. (2017). A Two-Dimensional Biodegradable Niobium Carbide (MXene) for Photothermal Tumor Eradication in NIR-I and NIR-II Biowindows. J. Am. Chem. Soc..

[163] Grobmyer S.R., Gutwein L.G., Singh A.K., Hahn M.A., Rule M.C., Knapik J.A., Moudgil B.M., Brown S.C. (2012). Fractionated photothermal antitumor therapy with multidye nanoparticles. Int. J. Nanomed..

[164] Hsiao C.-W., Chuang E.-Y., Chen H.-L., Wan D., Korupalli C., Liao Z.-X., Chiu Y.-L., Chia W.-T., Lin K.-J., Sung H.-W. (2015). Photothermal tumor ablation in mice with repeated therapy sessions using NIR-absorbing micellar hydrogels formed in situ. Biomaterials.

[165] Ren Y., Qi H., Chen Q., Ruan L. (2017). Thermal dosage investigation for optimal temperature distribution in gold nanoparticle enhanced photothermal therapy. Int. J. Heat Mass Transf..

[166] Simón M., Nørregaard K., Jørgensen J.T., Oddershede L.B., Kjaer A. (2019). Fractionated photothermal therapy in a murine tumor model: Comparison with single dose. Int. J. Nanomed..

[167] Hirsch L.R., Stafford R.J., Bankson J.A., Sershen S.R., Rivera B., Price R.E., Hazle J.D., Halas N.J., West J.L. (2003). Nanoshell-mediated near-infrared thermal therapy of tumors under magnetic resonance guidance. Proc. Natl. Acad. Sci. USA.

[168] Chen Q., Xu L., Liang C., Wang C., Peng R., Liu Z. (2017). Photothermal therapy with immune-adjuvant nanoparticles together with checkpoint blockade for effective cancer immunotherapy. Nat. Commun..

[169] Piao J.-G., Gao F., Yang L. (2016). Acid-Responsive Therapeutic Polymer for Prolonging Nanoparticle Circulation Lifetime and Destroying Drug-Resistant Tumors. ACS Appl. Mater. Interfaces.

[170] El-Sayed M.A., Ali M.R.K., Ibrahim I.M., Ali H.R., Selim S.A. (2016). Treatment of natural mammary gland tumors in canines and felines using gold nanorods-assisted plasmonic photothermal therapy to induce tumor apoptosis. Int. J. Nanomed..

[171] Sato K., Sato N., Xu B., Nakamura Y., Nagaya T., Choyke P.L., Hasegawa Y., Kobayashi H. (2016). Spatially selective depletion of tumor-associated regulatory T cells with near-infrared photoimmunotherapy. Sci. Transl. Med..

[172] Mooney R., Roma L., Zhao D., Van Haute D., Garcia E., Kim S.U., Annala A.J., Aboody K.S., Berlin J.M. (2014). Neural Stem Cell-Mediated Intratumoral Delivery of Gold Nanorods Improves Photothermal Therapy. ACS Nano.

[173] Gardner B., Matousek P., Stone N. (2019). Direct monitoring of light mediated hyperthermia induced within mammalian tissues using surface enhanced spatially offset Raman spectroscopy (T-SESORS). Analyst.

[174] Bagavathiappan S., Lahiri B.B., Saravanan T., Philip J., Jayakumar T. (2013). Infrared thermography for condition monitoring—A review. Infrared Phys. Technol..

[175] Levick A., Land D., Hand J. (2011). Validation of microwave radiometry for measuring the internal temperature profile of human tissue. Meas. Sci. Technol..

[176] Feddersen T.V., Hernandez-Tamames J.A., Franckena M., Van Rhoon G.C., Paulides M.M. (2021). Clinical Performance and Future Potential of Magnetic Resonance Thermometry in Hyperthermia. Cancers.

[177] West C.L., Doughty A.C., Liu K., Chen W.R. (2019). Monitoring tissue temperature during photothermal therapy for cancer. J. Bio-X Res..

[178] Bruggmoser G., Bauchowitz S., Canters R., Crezee J., Ehmann M., Gellermann J., Lamprecht U., Lomax N., Messmer M.B., Ott O. (2012). Guideline for the clinical application, documentation and analysis of clinical studies for regional deep hyperthermia. Strahlenther. Onkol..

[179] van der Zee J., Peer-Valstar J.N., Rietveld P.J., de Graaf-Strukowska L., van Rhoon G.C. (1998). Practical limitations of interstitial thermometry during deep hyperthermia. Int. J. Radiat. Oncol. Biol. Phys..

[180] Wust P., Gellermann J., Harder C., Tilly W., Rau B., Dinges S., Schlag P., Budach V., Felix R. (1998). Rationale for using invasive thermometry for regional hyperthermia of pelvic tumors. Int. J. Radiat. Oncol..

[181] Hynynen K., McDannold N., Mulkern R.V., Jolesz F.A. (2000). Temperature monitoring in fat with MRI. Magn. Reson. Med..

[182] Ghita A., Matousek P., Stone N. (2018). Sensitivity of Transmission Raman Spectroscopy Signals to Temperature of Biological Tissues. Sci. Rep..

[183] Lanka P., Francis K.J., Kruit H., Farina A., Cubeddu R., Sekar S.K.V., Manohar S., Pifferi A. (2021). Optical signatures of radiofrequency ablation in biological tissues. Sci. Rep..

[184] Jaywant S.M., Wilson B.C., Patterson M.S., Lilge L.D., Flotte T.J., Woolsey J., McCulloch C. (1993). Temperature-dependent changes in the optical absorption and scattering spectra of tissues: Correlation with ultrastructure. Laser-Tissue Interaction IV.

[185] van der Meer F.J., Faber D.J., Cilesiz I.F., van Gemert M.J., van Leeuwen T.G. (2006). Temperature-dependent optical properties of individual vascular wall components measured by optical coherence tomography. J. Biomed. Opt..

[186] Huang B., Tian Y., Li Z., Gao S., Li Z. (2007). Temperature measurement from the intensity ratio of the Raman-scattering lines in carbon tetrachloride constituting the liquid core of an optical fiber. Instrum. Exp. Tech..

[187] Mosca S., Dey P., Salimi M., Gardner B., Palombo F., Stone N., Matousek P. (2021). Estimating the Reduced Scattering Coefficient of Turbid Media Using Spatially Offset Raman Spectroscopy. Anal. Chem..

[188] Mosca S., Dey P., Salimi M., Palombo F., Stone N., Matousek P. (2020). Non-invasive depth determination of inclusion in biological tissues using spatially offset Raman spectroscopy with external calibration. Analyst.

[189] Mosca S., Dey P., Salimi M., Gardner B., Palombo F., Stone N., Matousek P. (2021). Spatially Offset Raman Spectroscopy—How Deep?. Anal. Chem..

[190] Gardner B., Matousek P., Stone N., Stone N. (2016). Temperature Spatially Offset Raman Spectroscopy (T-SORS): Subsurface Chemically Specific Measurement of Temperature in Turbid Media Using Anti-Stokes Spatially Offset Raman Spectroscopy. Anal. Chem..

[191] Gardner B., Stone N., Matousek P. (2016). Non-invasive chemically specific measurement of subsurface temperature in biological tissues using surface-enhanced spatially offset Raman spectroscopy. Faraday Discuss..

[192] Xie H., Stevenson R., Stone N., Hernandez-Santana A., Faulds K., Graham D. (2012). Tracking Bisphosphonates through a 20 mm Thick Porcine Tissue by Using Surface-Enhanced Spatially Offset Raman Spectroscopy. Angew. Chem. Int. Ed..

[193] Quintanilla M., García I., de Lázaro I., García-Alvarez R., Henriksen-Lacey M., Vranic S., Kostarelos K., Liz-Marzán L.M. (2019). Thermal monitoring during photothermia: Hybrid probes for simultaneous plasmonic heating and near-infrared optical nanothermometry. Theranostics.

[194] Yu Z.F., Shi J.P., Li J.L., Li P.H., Zhang H.W. (2018). Luminescence enhancement of CaF_2_: Nd^3+^ nanoparticles in the second near-infrared window for in vivo imaging through Y^3+^ doping. J. Mater. Chem. B.

